# Eclampsia Incidence, Management and Outcomes Across Multi‐Country Surveillance Cohorts: Individual Participant Data Meta‐Analysis

**DOI:** 10.1111/1471-0528.70216

**Published:** 2026-03-26

**Authors:** Silvia Salvi, Donatella Mandolini, Kitty W. M. Bloemenkamp, Catherine Deneux‐Tharaux, Hilde Marie Engjom, Lachmi R. Kodan, Diane Korb, Alexandra Krištúfková, Kari Klungsøyr, Ann Langedock, Camilla Tjønneland Mentzoni, Liam McCullough, Rema Ramakrishnan, T. P. (Timme) Schaap, Aurélien Seco, Griet Vandenberghe, Kim J. C. Verschueren, Serena Donati, INOSS Group (International Network of Obstetric Survey Systems)

**Affiliations:** ^1^ National Centre for Disease Prevention and Health Promotion, Istituto Superiore di Sanità—Italian National Institute of Health Rome Italy; ^2^ Dipartimento Scienze Della Vita e Sanità Pubblica Università Cattolica del Sacro Cuore, Fondazione Policlinico Universitario A. Gemelli IRCCS Rome Italy; ^3^ Department of Obstetrics, Birth Centre Wilhelmina Children Hospital, Division Women and Baby University Medical Centre Utrecht Utrecht the Netherlands; ^4^ Université Paris Cité, CRESS, Obstetrical Perinatal and Pediatric Lifelong Epidemiology Research Team, OPPaLE, INSERM, INRAE Paris France; ^5^ Division of Public Health and Prevention, Department of Health Promotion Norwegian Institute of Public Health Bergen Norway; ^6^ Department of Obstetrics Academic Hospital of Paramaribo Paramaribo Suriname; ^7^ First Department of Obstetrics and Gynaecology of Faculty of Medicine Comenius University and University Hospital Bratislava Slovakia; ^8^ Department of Global Public Health and Primary Care University of Bergen Bergen Norway; ^9^ Department of Obstetrics and Gynecology Groeninge Hospital Kortrijk Belgium; ^10^ National Perinatal Epidemiology Unit, Nuffield Department of Population Health University of Oxford Oxford UK; ^11^ Oxford NIHR Biomedical Research Centre Oxford UK; ^12^ Belgian Obstetric Surveillance System Belgium; ^13^ Department of Obstetrics and Gynecology Ghent University Hospital Ghent Belgium

**Keywords:** eclampsia, magnesium sulphate, maternal health, maternal mortality, perinatal care

## Abstract

**Objective:**

To estimate the incidence of eclampsia, characterise maternal and perinatal profiles, and document outcomes across seven countries within the International Obstetric Survey System and identify inter‐country differences that may improve maternal and perinatal care.

**Design:**

Multi‐country analysis of population‐based cohort data.

**Setting:**

Six high‐income countries (Belgium, France, Italy, the Netherlands, Norway, Slovakia) and one upper‐middle‐income country (Suriname).

**Population:**

All women admitted with eclampsia in participating countries between 2012 and 2019.

**Methods:**

Individual participant data meta‐analysis.

**Main Outcome Measures:**

Incidence of eclampsia, maternal demographics, pregnancy characteristics, clinical management, mode of delivery and maternal and perinatal outcomes.

**Results:**

615 cases of eclampsia were notified resulting in a pooled incidence of eclampsia of 2.2 per 10,000 deliveries in high‐income countries and 36.6 per 10,000 deliveries in Suriname. About 42% of women were diagnosed with preeclampsia before seizure onset and one‐third experienced their first seizure postpartum. Hypertension was the most reported clinical sign (91.1%). Most women were treated with magnesium sulphate (91.1%) and antihypertensive medications (89.8%). Caesarean section was performed in 72.7% of cases. About 53% of births was preterm with most of them linked to antepartum cases. Maternal and neonatal deaths were rare but more frequent in Suriname.

**Conclusions:**

The declining incidence of eclampsia in Europe may be attributed to enhanced management, supported by ongoing audits and confidential enquiries; however, potential ascertainment bias limits causal interpretation. Global efforts remain crucial to promote awareness, timely prevention and implement standardised management guidelines for eclampsia across all settings.

## 1 Introduction

Hypertensive disorders in pregnancy (HDP), a major global health concern, are associated with increased maternal and perinatal mortality, particularly in low‐middle‐income countries (LMICs) [1]. Eclampsia, characterised by sudden and generalised seizures, is a critical complication of pregnancy that affects 0.8% of HDP pregnancies and is often preventable with proper care [2]. The Global Burden of Disease data show a decline in the overall burden of HDP but lack specific estimates for eclampsia [3].

The incidence of eclampsia varies from 2 to 10 per 10,000 deliveries in high‐income countries (HICs) and 50 to 151 per 10,000 deliveries in low‐ and middle‐income countries (LMICs) [4]. Possible reasons for these disparities include variations in risk factors, antenatal care, management of severe preeclampsia, study design, case definitions, case identification methods and reliance on hospital‐based data. The high rates in LMICs and disparities within and among HICs underscore the need for better preventive care and timely management [5, 6].

Since 2010, the International Network of Obstetric Survey Systems (INOSS) has coordinated multi‐country, population‐based studies on rare and severe maternal morbidity through Enhanced Obstetric Surveillance Systems (EOSS) [7]. EOSS's critical case reviews, including audits and confidential enquiries, have facilitated cross‐country comparisons and research quality.

We aimed to synthesise population‐based data from multiple INOSS HICs and one upper‐middle‐income country (UMIC) to estimate the incidence of eclampsia, characterise maternal and antenatal profiles, investigate clinical and seizure characteristics, describe management practices and mode of delivery, document maternal and perinatal outcomes and assess inter‐country differences to improve maternal and perinatal care.

## 2 Methods

This individual participant data (IPD) meta‐analysis used population‐based data from cohort studies within INOSS from 2012 to 2019. Data were collected nationwide, except in France and Italy, where multi‐regional studies covered 20% and 75% of national births, respectively [8, 9]. In Italy, France, Slovakia, Suriname and the Netherlands, informed consent was not required as data were part of EOSS activities and in Norway and Belgium the study was exempt from individual consent.

Preeclampsia was diagnosed as hypertension (≥ 140/90 mmHg on two occasions, at least 4 h apart) with proteinuria (≥ 0.3 g in a 24‐h collection) after 20 weeks' gestation for cases identified from 2012 to 2014, and according to the 2014 International Society for the Study of Hypertension in Pregnancy guidelines for data after 2014 [10].

Eclampsia was defined as seizures during pregnancy or postpartum, not attributable to other causes. Detailed definitions and the distribution of findings across diagnostic criteria for eclampsia by country are provided in Tables S1 and S2. Eclampsia cases were prospectively identified in all countries except Slovakia, where cases were reported through the National Case Register and Slovak Obstetric Survey System (SOSS), followed by retrieval of information from clinical records. Belgium, France, Italy, the Netherlands and Suriname utilised their respective EOSS for case reporting, while Norway used mandatory notifications to the Medical Birth Registry of Norway (MBRN) [8, 9, 11, 12, 13].

The source population (*n* = 2,611,553) included all women who delivered live or still births beyond 22 weeks' gestation during the study period in the participating maternity units. Details on the source populations and methodologies of the reference studies are provided in Tables S3 and S4 and in previous publications [5, 8, 9, 11, 12, 14, 15, 16].

Details of variables for maternal demographics, pregnancy characteristics, clinical management, delivery mode and maternal and perinatal outcomes are provided in Table S5.

A common Case Report Form (CRF), developed by a multidisciplinary team and revised by country experts, was used for multinational data collection. All countries completed the CRF through medical record review and audits were conducted in all countries except Belgium and Norway. In Norway, the MBRN provided ante, intra and postnatal data via a standard birth notification form. Though the Norwegian data lacked some clinical details, all notified cases of eclampsia were verified.

Country‐level data were stored in a central database, coordinated by the Italian Obstetric Surveillance System. Each country obtained approval from local Ethics Committees and Data Protection Officers and signed a Data Transfer Agreement for anonymous aggregated data transfer. To minimise bias, countries were asked to recheck source data for incompleteness or inconsistency. France, Italy and Suriname validated reported cases using the National Hospital Discharge database (NHDd) and diagnostic codes (ICD‐9‐CM: 642.6; ICD‐10: O15). Discrepancies led to a review with maternity units to verify data accuracy, addressing over/under‐reporting and missing information.

## 3 Statistical Analysis

The sample size was based on the number of cases reported during the study periods in participating countries, with no formal power calculation performed. Eclampsia incidence per 10,000 deliveries was estimated separately for HICs and Suriname. Descriptive statistics included frequencies and percentages for categorical variables and medians (IQR) or means (SD) for continuous variables. Percentages excluded missing values. Variables with > 25% missing data were reported only as absolute numbers.

A one‐stage IPD meta‐analysis was conducted using Stata's ‘metapreg’ package for meta‐analysis of binomial data with a hierarchical random effects model, using binomial distribution and Wilson's confidence intervals for small proportions [17, 18]. The metapreg command enabled a one‐stage meta‐analysis of proportions using country‐specific numerators and denominators. We computed pooled incidence of eclampsia per 10,000 deliveries in HICs and estimated pooled proportions for clinical characteristics, information on eclampsia diagnosis, prevention and management, timing of delivery and maternal and perinatal outcomes (Table S5). Meta‐analysis was not performed if data from ≥ 3 countries were unavailable or when missing data in one country exceeded 25%. Heterogeneity across countries was assessed using the *I* ^2^ statistic, τ^2^ and prediction intervals.

Pooled analyses were conducted by including and then excluding Suriname for selected variables—namely number of seizures (one or ≥ 3), maternal higher‐level monitoring, neonatal intensive care unit (NICU) admission and PRES—to account for potential differences in healthcare systems and organisation of care across settings. A sensitivity analysis excluding data from Belgium and France was also performed to assess the impact of older data. Differences in pooled proportions were evaluated for indicators potentially sensitive to temporal changes—namely incidence, preeclampsia diagnosis, number of seizures, antihypertensive treatment, MgSO_4_ treatment, anticonvulsants administration, mode of delivery, preterm delivery, HELLP syndrome and intrauterine foetal death.

Statistical analyses were performed using StataSE version 18.

## 4 Results

From 2012 to 2019, 615 cases of eclampsia were diagnosed in the six HICs resulting in a pooled incidence of 2.2 per 10,000 deliveries (95% CI 1.5–3.5, *τ* ^2^ = 0.10, predictive interval 1.3–3.4); the incidence ranged from 1.5 in Italy to 3.8 in Norway (Figure S1). The incidence in Suriname was 36.6 per 10,000 deliveries. About 42% women were diagnosed with preeclampsia before seizures (95% CI 24.2–61.4, *τ* ^2^ = 0.72, predictive interval 6.7–86.2) (Figure S2), with the highest proportion in France (64.7%, 33/51) and the lowest Suriname (15.3%, 11/72) and in Italy (20.2%, 21/104).

Across all countries, most women with eclampsia were nulliparous. Adolescents and non‐native women were overrepresented compared to the general population. Although rare, multiple pregnancies were more frequent among eclampsia cases compared to the source population in each country, except in the Netherlands and Suriname (Table S6).

### 4.1 Clinical and Seizure Characteristics

Table 1 shows details of the premonitory symptoms and signs. Headache was the most common symptom (55.7%, 95% CI 39.9–69.3, *τ* ^2^ = 0.20, predictive interval 32.3–77.2), followed by epigastric pain (26.6%, 95% CI 12.0–51.6, *τ* ^2^ = 0.85, predictive interval 1.4–86.3) (Figures S3 and S4).

**TABLE 1:** Clinical characteristics.

	Belgium (*n* = 64)	France (*n* = 51)	Italy (*n* = 109)	Netherlands (*n* = 88)	Norway (*n* = 174)	Slovakia (*n* = 57)	Suriname (*n* = 72)	Pooled estimates, % (95% CI)
Diagnosis of preeclampsia								
Yes	22	33 (64.7%)	21 (20.2%)	30 (39.5%)	109 (62.6%)	27 (49.1%)	11 (15.3%)	41.8 (24.2–61.4)
No	17	18 (35.3%)	83 (79.8%)	46 (60.5%)	65 (37.4%)	28 (50.9%)	61 (84.7%)	
*Missing*	*25* ^a^	*0 (0.0%)*	*5 (4.6%)*	*12 (13.6%)*	*0 (0.0%)*	*2 (3.5%)*	*0 (0.0%)*	
Premonitory symptoms: headache								
Yes	37 (71.2%)	2	47 (43.1%)	48 (70.6%)	NA	27 (47.4%)	33 (47.8%)	55.7 (39.9–69.3)
No	15 (28.8%)	17	62 (56.9%)	20 (29.4%)	NA	30 (52.6%)	36 (52.2%)	
*Missing*	*12 (18.8%)*	*32* ^a^	*0 (0.0%)*	*20 (22.7%)*	*NA*	*0 (0.0%)*	*3 (4.2%)*	
Premonitory symptoms: epigastric pain								
Yes	17 (35.4%)	8	25 (22.9%)	36 (53.7%)	NA	9 (15.8%)	4 (5.8%)	26.6 (12.0–51.6)
No	31 (64.6%)	11	84 (77.1%)	31 (46.3%)	NA	48 (84.2%)	65 (94.2%)	
*Missing*	*16 (25.0%)*	*32* ^a^	*0 (0.0%)*	*21 (23.9%)*	*NA*	*0 (0.0%)*	*3 (4.2%)*	
Premonitory symptoms: nausea/vomiting								
Yes	NA	4	15 (13.8%)	42 (60.0%)	NA	17 (29.8%)	15 (21.7%)	—
No	NA	15	94 (86.2%)	28 (40.0%)	NA	40 (70.2%)	54 (78.3%)	
*Missing*	*NA*	*32* ^a^	*0 (0.0%)*	*18 (20.5%)*	*NA*	*0 (0.0%)*	*3 (4.2%)*	
Systolic BP < 24 h before the fit (mean)	162.4 (28.4)	145.9 (28.0)	151.4 (31.0)	155.1 (23.4)	NA	NA	158.0 (21.0)	—
Diastolic BP < 24 h before the fit (mean)	96.1 (15.7)	88.5 (18.9)	97.8 (19.1)	97.9 (16.9)	NA	NA	99.0 (17.0)	—
Systolic BP at the fit (mean)	180.4 (27.9)	174.3 (27.5)	177.2 (25.8)	160.5 (24.0)	NA	181.8 (25.2)	164.0 (21.0)	—
Diastolic BP at the fit (mean)	106.3 (14.0)	104.3 (16.6)	109.7 (18.4)	95.8 (15.6)	NA	117.3 (22.3)	105.0 (14.0)	—

*Note:* Data: *n* (%), mean (SD).

Abbreviations: BP, blood pressure; NA, not available.

^a^ Variable excluded due to > 25% missing data (italic).

Mean blood pressure exceeded 145/88 mmHg 24 h before the seizure and was above 160/96 mmHg at seizure onset, across all countries (Table 1). Hypertension accompanied seizures in most cases (91.1%, 95% CI 73.0–94.9, *τ* ^2^ = 0.11, predictive interval 81.3–96.3) (Figure S5), while thrombocytopenia and increased alanine aminotransferase (ALT) or aspartate aminotransferase (AST) were less frequently reported (Figures S6 and S7).

Table 2 summarises the characteristics of eclampsia by timing from delivery. Two‐thirds of women had seizures ante‐ or intrapartum, while one‐third (32.0%, 95% CI 28.6–36.1, *τ* ^2^ = 0 < 0.0001, predictive interval 27.3–37.2) of seizures occurred postpartum (Figure S8a–c). Fewer than 3% of postpartum cases occurred more than 7 days after delivery. Antepartum seizures occurred at a mean gestational age of 32–34 weeks, while intrapartum seizures occurred at 36–40 weeks, with Belgium reporting the lowest gestational age at seizure occurrence. The median time from the first seizure to delivery was shortest for intrapartum cases. For antepartum seizures, Slovakia had the shortest median interval (40 min), while the Netherlands among HICs (315 min) and Suriname (1650 min) reported the longest. In HICs, most women experienced a single fit (66.2%, 95% CI 61.0–71.6, *τ* ^2^ < 0.0001, predictive interval 53.5–76.9) (Figure S9a); in Suriname, 40.3% (29/72) of women had one fit. In HICs with available data, 6 to 12.5% of the women experienced three or more fits (9.9%, 95% CI 7.1–14.1, *τ* ^2^ < 0.0001, predictive interval 4.5–20.3) (Figure S11a), while in Suriname this proportion was 25% (18/72). A first fit at home was more common in the Netherlands (34.2%, 28/82) and Suriname (34.7%, 25/72).

**TABLE 2:** Fit characteristics.

	Belgium (*n* = 64)	France (*n* = 51)	Italy (*n* = 109)	Netherlands (*n* = 88)	Norway (*n* = 174)	Slovakia (*n* = 57)	Suriname (*n* = 72)	Pooled estimates, % (95% CI)
Timing of first fit								
Antepartum	39 (60.9%)	23 (45.1%)	55 (52.4%)	37 (45.1%)	62 (35.6%)	28 (62.2%)	39 (54.2%)	49.9 (40.8–58.7)
Intrapartum	6 (9.4%)	8 (15.7%)	11 (10.5%)	19 (23.2%)	59 (33.9%)	3 (6.7%)	14 (19.4%)	17.4 (10.6–29.5)
Postpartum	19 (29.7%)	20 (39.2%)	39 (37.1%)	26 (31.7%)	53 (30.5%)	14 (31.1%)	19 (26.4%)	32.0 (28.6–36.1)
*Missing*	*0 (0.0%)*	*0 (0.0%)*	*4 (3.7%)*	*6 (6.8%)*	*0 (0.0%)*	*12 (21.1%)*	*0 (0.0%)*	
Number of fits								
1	46 (71.9%)	26 (54.2%)	64 (64.6%)	58 (70.7%)	NA	NA	29 (40.3%)	66.2 (61.0–71.6)^a^
2	14 (21.9%)	16 (33.3%)	23 (23.2%)	17 (20.7%)	NA	NA	25 (34.7%)	26.1 (19.0–49.4)
≥ 3	4 (6.3%)	6 (12.5%)	12 (12.1%)	7 (8.5%)	NA	NA	18 (25.0%)	9.9 (7.1–14.1)^a^
*Missing*	*0 (0.0%)*	*3 (5.9%)*	*10 (9.2%)*	*6 (6.8%)*	*NA*	*NA*	*0 (0.0%)*	
Location of first fit								
Hospital	44 (69.8%)	38 (77.6%)	83 (78.3%)	54 (65.9%)	NA	36 (73.5%)	47 (65.3%)	71.7 (49.7–78.8)
Home	16 (25.4%)	9 (18.4%)	19 (17.9%)	28 (34.1%)	NA	12 (24.5%)	25 (34.7%)	25.9 (19.4–35.3)
Other	3 (4.8%)	2 (4.1%)	4 (3.8%)	0 (0.0%)	NA	1 (2.0%)	0 (0.0%)	—
*Missing*	*1 (1.6%)*	*2 (3.9%)*	*3 (2.8%)*	*6 (6.8%)*	*NA*	*8 (14.0%)*	*0 (0.0%)*	
Gestational age at the first fit (wks)								
Antepartum (mean)	31.6 (3.8)	32.7 (4.3)	34.0 (4.1)	33.3 (3.3)	33.7 (4.3)	34.3 (4.1)	33.0 (4.0)	—
Intrapartum (mean)	36.2 (5.7)	38.2 (3.4)	39.1 (1.8)	39.5 (1.4)	37.19 (4.0)	39.3 (0.6)	37.0 (3.0)	—
First fit—delivery interval (min)								
Antepartum (median)	92.5 (30–350)	64 (31–144)	62 (20–172)	315 (190–728)	NA	40 (16–115)	1650 (900–2460)	—
Intrapartum (median)	39 (13–55)	28 (16–37)	10 (5–55)	39 (19–107)	NA	NA	90 (30–120)	—
Delivery—first fit interval (min)								
Postpartum cases (median)	1458 (355–8017)	336 (179–886)	477 (245–806)	234 (31–1044)	NA	282 (85–760)	210 (30–840)	—

*Note:* Data: *n* (%), mean (SD), median (IQR).

Abbreviations: min, minutes; NA, not available; wks, weeks.

^a^ Restricted to European countries.

### 4.2 Management Practices and Mode of Delivery

Table 3 summarises the prevention and treatment of seizures and mode of delivery. MgSO_4_ prophylaxis for preeclampsia was highest in Suriname (72.7%, 8/11) and lowest in France (3.0%, 1/33). The pooled proportion of women who received MgSO_4_ treatment was 91.1% (95% CI 72.2–96.0, *τ* ^2^ = 0.79, predictive interval 51.7–99.4) with all cases in the Netherlands having received MgSO_4_ (Figure S14). The pooled proportion of antihypertensive medications use was 89.8% (95% CI 65.7–96.3, *τ* ^2^ = 1.30, predictive interval 21.1–99.9) (Figure S15). The pooled proportion of other anticonvulsants administration was 43.1% (95% CI 29.9–56.9, *τ* ^2^ = 0.23, predictive interval 23.3–64.6), ranging from 26.3% (15/57) in Slovakia to 62.7% (37/59) in Belgium (Figure S16).

**TABLE 3:** Management and mode of delivery.

	Belgium (*n* = 64)	France (*n* = 51)	Italy (*n* = 109)	Netherlands (*n* = 88)	Norway (*n* = 174)	Slovakia (*n* = 57)	Suriname (*n* = 72)	Pooled estimates, % (95% CI)
MgSO_4_ prophylaxis among preeclamptic women								
Yes	NA	1 (3.0%)	6 (28.6%)	3 (10.0%)	NA	NA	8 (72.7%)	—
No	NA	32 (97.0%)	15 (71.4%)	27 (90.0%)	NA	NA	3 (27.3%)	
*Missing*	*NA*	*0 (0.0%)*	*0 (0.0%)*	*0 (0.0%)*	*NA*	*NA*	*0 (0.0%)*	
Antihypertensive treatment								
Yes	54 (93.1%)	44 (86.3%)	95 (87.2%)	61 (74.4%)	NA	57 (100.0%)	59 (98.3%)	89.8 (65.7–96.3)
No	4 (6.9%)	7 (13.7%)	14 (12.8%)	21 (25.6%)	NA	0 (0.0%)	1 (1.7%)	
*Missing*	*6 (9.4%)*	*0 (0.0%)*	*0 (0.0%)*	*6 (6.8%)*	*NA*	*0 (0.0%)*	*12 (16.7%)*	
MgSO_4_ treatment								
Yes	59 (93.7%)	42 (82.4%)	97 (89.0%)	82 (100.0%)	NA	47 (83.9%)	69 (95.8%)	91.1 (72.2–96.0)
No	4 (6.3%)	9 (17.6%)	12 (11.0%)	0 (0.0%)	NA	9 (16.1%)	3 (4.2%)	
*Missing*	*1 (1.6%)*	*0 (0.0%)*	*0 (0.0%)*	*6 (6.8%)*	*NA*	*1 (1.8%)*	*0 (0.0%)*	
Other anticonvulsants								
Yes	37 (62.7%)	20 (39.2%)	60 (55.0%)	24 (30.0%)	NA	15 (26.3%)	27 (44.3%)	43.1 (29.9–56.9)
No	22 (37.3%)	31 (60.8%)	49 (45.0%)	56 (70.0%)	NA	42 (73.7%)	34 (55.7%)	
*Missing*	*5 (7.8%)*	*0 (0.0%)*	*0 (0.0%)*	*8 (9.1%)*	*NA*	*0 (0.0%)*	*11 (15.3%)*	
Gestational Age at delivery (wks)								
Antepartum (mean)	31.6 (3.8)	33.1 (4.3)	34.0 (NA)	33.4 (3.3)	33.7 (4.3)	34.3 (4.1)	33.0 (4.0)	—
Intrapartum (mean)	36.2 (5.7)	38.2 (3.4)	39.1 (NA)	39.5 (1.4)	37.2 (4.0)	39.3 (0.6)	37.0 (3.0)	—
Postpartum (mean)	38.5 (2.8)	37.3 (3.2)	36.9 (NA)	38.5 (2.0)	37.3 (3.0)	35.8 (3.1)	37.0 (2.0)	—
Mode of delivery: CS								
All cases	49 (76.6%)	36 (70.6%)	89 (81.7%)	55 (62.5%)	115 (66.1%)	51 (89.5%)	46 (63.9%)	72.7 (61.2–80.6)
Antepartum	36 (73.5%)	23 (63.9%)	55 (62.5%)	32 (65.3%)	53 (46.1%)	27 (69.2%)	29 (64.4%)	62.5 (52.8–70.6)
Intrapartum	5 (10.2%)	5 (13.9%)	9 (10.2%)	9 (18.4%)	43 (37.4%)	2 (5.1%)	11 (24.4%)	17.4 (9.8–31.5)
Postpartum	8 (16.3%)	8 (22.2%)	24 (27.3%)	8 (16.3%)	19 (16.5%)	10 (25.6%)	5 (11.1%)	19.4 (14.7–36.8)
*Missing*	*0 (0.0%)*	*0 (0.0%)*	*1 (1.1%)*	*6 (10.9%)*	*0 (0.0%)*	*12 (23.5%)*	*1 (2.2%)*	
Mode of delivery: urgent/emergency CS								
All cases	38 (59.4%)	34 (66.7%)	84 (77.1%)	22	112 (64.4%)	NA	19 (26.4%)	59.1 (37.8–75.5)
Antepartum	30 (78.9%)	23 (67.6%)	55 (66.3%)	4	52 (46.4%)	NA	10 (52.6%)	62.1 (46.3–74.4)
Intrapartum	4 (10.5%)	5 (14.7%)	9 (10.8%)	9	42 (37.5%)	NA	7 (36.8%)	21.6 (10.8–41.0)
Postpartum	4 (10.5%)	6 (17.6%)	19 (22.9%)	3	18 (16.1%)	NA	2 (10.5%)	—
*Missing*	*0 (0.0%)*	*0 (0.0%)*	*1 (1.2%)*	*6* ^a^	*0 (0.0%)*	*NA*	*0 (0.0%)*	

*Note:* Data: *n* (%), mean (SD).

Abbreviations: CS, Caesarean Section; MgSO_4_, Magnesium Sulfate; NA, not available.

^a^ Variable excluded due to > 25% missing data.

In all countries, gestational age at delivery ranged from 32 to 34 weeks for antepartum cases and from 36 to 39 weeks for intrapartum and postpartum cases. About 73% of eclampsia cases had a caesarean delivery (95% CI 61.2–80.6, *τ* ^2^ = 0.18, predictive interval 58.8–84.3) (Figure S17); 62.5% of these were antepartum (95% CI 52.8–70.6, *τ* ^2^ = 0.08, predictive interval 51.5–72.6) and 17.4% were intrapartum (95% CI 9.8–31.5, *τ* ^2^ = 0.41, predictive interval 4.9–40.2) (Figure S17a,b). Urgent/emergency caesarean deliveries varied by country and seizure timing (Table 3). The pooled proportion of urgent/emergency caesarean delivery was 59.1% (95% CI 37.8–75.5, *τ* ^2^ = 0.52, predictive interval 17.0–91.4) with the majority performed antepartum (62.1%, 95% CI 46.3–74.4, *τ* ^2^ = 0.18, predictive interval 39.1–81.3) (Figure S18).

### 4.3 Maternal Outcomes

Table 4 shows maternal outcomes from all countries except the Netherlands.

**TABLE 4:** Maternal and perinatal outcomes.

	Belgium (*n* = 64)	France (*n* = 51)	Italy (*n* = 109)	Netherlands (*n* = 88)	Norway (*n* = 174)	Slovakia (*n* = 57)	Suriname (*n* = 72)	Pooled estimates, % (95% CI)
**Maternal outcomes**								
Preterm deliveries (< 37 weeks)								
All cases	41 (64.1%)	27 (52.9%)	50 (45.9%)	47 (53.4%)	78 (44.8%)	37 (64.9%)	36 (50.0%)	52.6 (45.1–60.0)
Antepartum	37 (90.2%)	20 (74.1%)	38 (76.0%)	32 (88.9%)	42 (54.5%)	20 (69.0%)	29 (80.6%)	76.0 (60.5–84.6)
Intrapartum	1 (2.4%)	2 (7.4%)	1 (2.0%)	0 (0.0%)	21 (27.3%)	0 (0.0%)	2 (5.6%)	2.7 (0.7–20.5)
Postpartum	3 (7.3%)	5 (18.5%)	11 (22.0%)	4 (11.1%)	14 (18.2%)	9 (31.0%)	5 (13.9%)	17.2 (12.2–71.0)
*Missing*	*0 (0.0%)*	*0 (0.0%)*	*0 (0.0%)*	*11 (23.4%)*	*1 (1.3%)*	*8 (21.6%)*	*0 (0.0%)*	
Higher‐level monitoring admission								
Yes	47 (75.8%)	34 (66.7%)	68 (62.4%)	NA	43	37 (64.9%)	36 (50.0%)	66.7 (61.3–72.2)^b^
No	15 (24.2%)	17 (33.3%)	41 (37.6%)	NA	0	20 (35.1%)	36 (50.0%)	
*Missing*	*2 (3.1%)*	*0 (0.0%)*	*0 (0.0%)*		*131* ^a^	*0 (0.0%)*	*0 (0.0%)*	
HELLP Syndrome	12 (18.8%)	9 (17.6%)	15 (13.8%)	11 (12.5%)	16 (9.2%)	4 (7.0%)	8 (11.1%)	12.2 (10.0–15.2)
PRES	4 (6.3%)	6 (11.8%)	15 (13.8%)	NA	0 (0.0%)	0 (0.0%)	3 (4.2%)	6.2 (0.8–41.3)
Maternal death	1 (1.6%)	0 (0.0%)	1 (0.9%)	1 (1.1%)	1 (0.6%)	1 (1.8%)	2 (2.8%)	—
**Perinatal outcomes**								
Total foetuses	(*n* = 66)	(*n* = 51)	(*n* = 113)	(*n* = 88)	(*n* = 185)	(*n* = 60)	(*n* = 73)	
Total live births	(*n* = 62)	(*n* = 47)	(*n* = 111)	(*n* = 87)	(*n* = 179)	(*n* = 60)	(*n* = 64)	
Birth weight (mean)	2048.0 (863)	2400.3 (385)	2421.0 (945)	2584.8 (798)	2617.0 (1043)	2206.7 (900)	2324.0 (826)	—
Apgar 5th < 7^c^								
Yes	13 (21.0%)	2 (4.3%)	13 (14.1%)	8 (10.3%)	25 (14.0%)	10 (16.9%)	10 (16.4%)	14.0 (11.6–17.3)
No	49 (79.0%)	44 (95.7%)	79 (85.9%)	70 (89.7%)	154 (86.0%)	49 (83.1%)	51 (83.6%)	
*Missing*	*0 (0.0%)*	*1 (2.1%)*	*19 (17.1%)*	*9 (10.3%)*	*0 (0.0%)*	*1 (1.7%)*	*3 (4.7%)*	
NICU admission^c^								
Yes	49 (79.0%)	18 (38.3%)	46 (42.2%)	20 (24.4%)	95 (53.1%)	28 (52.8%)	17 (27.4%)	48.2 (31.1–66.0)^b^
No	13 (21.0%)	29 (61.7%)	63 (57.8%)	62 (75.6%)	84 (46.9%)	25 (47.2%)	45 (72.6%)	
*Missing*	*0 (0.0%)*	*0 (0.0%)*	*2 (1.8%)*	*5 (5.7%)*	*0 (0.0%)*	*7 (11.7%)*	*2 (3.1%)*	
Perinatal death								
Intrauterine foetal death	4 (6.1%)	4 (7.8%)	2 (1.8%)	1 (1.1%)	6 (3.2%)	0 (0.0%)	9 (12.3%)	—
Neonatal death	0 (0.0%)	1 (2.0%)	1 (0.9%)	0 (0.0%)	1 (0.5%)	3 (5.0%)	2 (2.7%)	—
*Missing*	*0 (0.0%)*	*0 (0.0%)*	*0 (0.0%)*	*0 (0.0%)*	*0 (0.0%)*	*2 (3.3%)*	*0 (0.0%)*	

*Note:* Data: *n* (%), mean (SD).

Abbreviations: HELLP, Haemolysis, Elevated Liver enzymes and Low Platelets; NA, not available; NICU, neonatal intensive care unit; PRES, Posterior Encephalopathy Syndrome.

^a^ Variable excluded due to > 25% missing data.

^b^ Restricted to European countries.

^c^ Calculated on live births.

The pooled proportion of preterm deliveries was 52.6% (95% CI 45.1–60.0, *τ* ^2^ = 0.04, predictive interval 44.6–60.5) (Figure S19) and was mostly linked to antepartum cases (76.0% of preterm deliveries, 95% CI 60.5–84.6, *τ* ^2^ = 0.32, predictive interval 53.6–90.9) (Figure S19a). Maternal higher‐level monitoring admission was reported for 66.7% of women (95% CI 61.3–72.2, *τ* ^2^ < 0.0001, predictive interval 53.7–77.6) across the six HICs (Figure S20a) and 50.0% (36/72) in Suriname.

More than 12% (95% CI 10.0–15.2, *τ* ^2^ < 0.0001, predictive interval 9.2–16.0) of women with eclampsia had Haemolysis, Elevated Liver enzyme levels and Low Platelet (HELLP) and 6.2% (95% CI 0.8–41.3, *τ* ^2^ = 2.3, predictive interval 0.0–99.9) had Posterior Encephalopathy Syndrome (PRES) (Figures S21 and S22). Overall, seven maternal deaths were reported: one in each HIC except France and two in Suriname.

### 4.4 Perinatal Outcomes

Table 4 presents perinatal outcomes. The mean birthweight in each country mirrored the proportion of preterm deliveries, with Belgium reporting the lowest value. The proportion of intrauterine foetal death varied notably across countries with Suriname reporting the highest proportion (12.3%, 9/73). The pooled proportion of 5 min Apgar score < 7 was 14.0% (95% CI 11.6–17.3, *τ* ^2^ < 0.0001, predictive interval 10.7–18.2) (Figure S23) and about half of the infants were admitted to the NICU (48.2%; 95% CI 31.1–66.0, *τ* ^2^ = 0.48, predictive interval 16.3–81.8) across HICs (Figure S24a), while in Suriname it accounted for 27.4% (17/62). Neonatal deaths were rare, occurring in < 3% of the total foetuses in all countries except in Slovakia (5.0%, 3/60).

The sensitivity analysis, excluding Belgium and France, showed no significant differences in pooled proportions for any of the examined indicators, confirming the robustness of the main findings.

## 5 Discussion

### 5.1 Main Findings

The pooled incidence of eclampsia from 2012 to 2019 in the six HICs from the INOSS network was 2.2 per 10,000 deliveries and 36.6 per 10,000 in the South American UMIC, Suriname. Preeclampsia was diagnosed prior to seizure in 41.8% of the cases and 32.0% of cases occurred postpartum. While most countries had implemented MgSO_4_ as eclampsia treatment, use for prophylaxis appears to vary. Similarly, substantial variation was observed in the time from fit to delivery. Maternal and neonatal deaths were rare, with the highest numbers reported in Suriname.

### 5.2 Strengths and Limitations

The strengths of this study included standardised data collection, and an IPD meta‐analysis enabling robust cross‐country comparisons of incidence, heterogeneity, prevention and treatment. Most of the data were from national, population‐based cohorts with established case‐verification systems (e.g., ICD‐10 codes in hospital or national databases), enhancing data reliability. Study limitations included variation in national case definitions, but this is unlikely to have introduced selection bias given the clear clinical recognizability of eclampsia. Other limitations included significant heterogeneity that was observed for some outcomes and missing data that exceeded 25% for a few HICs. Lastly, data‐sharing regulations limited access to individual‐level data, requiring analyses based on aggregated country‐level data.

### 5.3 Interpretation

The estimated incidence of eclampsia was 2.2 cases per 10,000 deliveries across six HICs which is lower than previous national estimates of 4 to 6 per 10,000 deliveries [19, 20, 21]. Variation across Europe likely reflects improvement in clinical management and the availability of audit systems that facilitates the identification of care‐related gaps and opportunities for improvement. However, substantial heterogeneity suggests that ascertainment bias and differences in surveillance practices may also contribute to this variation. A study in France identified inadequate care as a main contributor to the high rate (2.8 per 10,000 deliveries) in 2012–2013, when eclampsia management was suboptimal [8]. In contrast, Italy, with the lowest rate (1.5 per 10,000 deliveries), likely benefited from recent awareness and training programs focused on HPDs [9, 22], which helped reduce the HPD‐specific maternal mortality ratio, from 1.06 per 100,000 live births in 2006–2012 to 0.62 in 2014–2018 [9]. Similarly, the Netherlands experienced a significant decrease in eclampsia incidence, from 6.2 per 10,000 deliveries in 2004–2006 to 1.8 in 2012–2019, alongside reduced perinatal mortality [12]. These success stories were driven by national EOSS initiatives, highlighting the importance of continued investment in their establishment and maintenance [23]. Norway has longstanding mandatory national case reporting and routine verification but lacks routine confidential enquiries. The relatively high Norwegian incidence (3.8 per 10,000 deliveries) and slower decline (from 5.0 per 10,000 in 1998–2000) suggest room for improvement in prevention and care, even in HICs with low maternal mortality [24]. Slovakia's eclampsia rate aligns with other HICs, but the Roma population accounted for most cases (26.6 per 10,000 deliveries) and faces challenges due to limited prenatal care and lower socio‐economic status [14].

The eclampsia rate in Suriname was 36.6 per 10,000 deliveries, a finding similar to other MICs but more than tenfold higher than the incidence in HICs [4]. The significant disparities in eclampsia incidence between HICs and MICs are concerning and remain poorly understood, particularly as women in MICs generally receive appropriate management for eclampsia in hospital facilities. The persistently high eclampsia rate may be influenced by risk factors such as ethnicity, nutritional status, adolescent pregnancies and limited access to intensive care facilities [25]. Addressing these issues, is crucial for reducing eclampsia rates and improving maternal and perinatal outcomes, as demonstrated by successful initiatives in other countries [5, 25].

The interpretation of premonitory symptoms is limited by their variability, scarce reporting and the influence of out‐of‐hospital cases, which accounted for 18%–35% of occurrences in our study. While maternal hypertension was the primary warning sign before the first seizure in 91.1% of cases, nearly 9% of eclampsia cases occurred in normotensive women. This highlights the importance for obstetricians to remain vigilant for atypical presentations, as delayed diagnosis and management can lead to worse outcomes [26]. In our study, in the six HICs, few women with diagnosed preeclampsia received prophylactic MgSO_4_. Although this suggests potential for improvement, assessing the effectiveness of MgSO_4_ prophylaxis is challenging due to the inability to determine successful seizure prevention. Moreover, these variations reflect the lack of international consensus on the use of MgSO_4_ prophylaxis in women with preeclampsia without severe features [27]. In nearly half of the cases, preeclampsia was not diagnosed before the seizure, highlighting the need for vigilant monitoring to detect early warning signs. This underscores the need for a prompt response to these signs, not only in women with known risk but also in those without a prior diagnosis of preeclampsia. Regarding eclampsia treatment, 1 in 10 women in our cohort did not receive MgSO_4_, increasing the risk of recurrence. The higher number of women with multiple seizures in France, where a smaller proportion of women received MgSO_4_, supports this. The use of antihypertensive medications was consistent across countries (89.8%), while the use of alternative anticonvulsants varied, from 26.3% in Slovakia to 62.7% in Belgium. This variation may reflect a higher proportion of out‐of‐hospital births, where first responders may rely on these drugs during seizures. Outdated clinical hospital practices or guidelines using other anticonvulsants could also contribute to this approach. Given the widespread use of these anticonvulsants and the associated risk of respiratory depression, further investigation is needed.

Although the overall proportion of caesarean deliveries was consistent with national averages, there were no notable differences in the proportions of urgent/emergency caesarean deliveries between HICs. Differences in urgent/emergency caesarean deliveries and the median time from first seizure to delivery between Suriname and HICs may reflect variations in healthcare access. Notably, one‐third of the first seizures occurred postpartum, challenging the assumption that birth resolves risks associated with preeclampsia. While immediate delivery was previously common for the management of eclampsia, current guidelines recommend a sequenced approach: prompt maternal stabilisation, MgSO_4_ infusion, blood pressure control, airway management and timely delivery [4, 28]. However, clear definitions for proper maternal stabilisation remain undefined [5].

While HELLP syndrome occurred at similar proportions, the diagnosis of PRES varied across countries, likely reflecting differences in access to cerebral imaging and case ascertainment. Frequent use of higher‐level monitoring reflects disease severity; however, its interpretation is limited by substantial variation in admission thresholds and organisational models across settings.

Perinatal outcomes in eclampsia are influenced by disparities in healthcare access and neonatal services, as seen in Suriname, and by the high prevalence of antepartum preterm deliveries, particularly in Belgium, where prematurity significantly affected Apgar scores and NICU admissions.

## 6 Conclusion

This large multi‐country individual participant data meta‐analysis, based on a source population of 2.6 million women giving birth, shows marked variation in eclampsia incidence among HICs and in Suriname. Fewer than half of women had a prior diagnosis of preeclampsia, one‐third of eclamptic seizures occurred postpartum, and 1 in 10 women did not receive MgSO_4_. Marked variation between countries in time from first seizure to delivery and mode of birth highlights differences in care pathways. These findings emphasise the need for renewed global attention to eclampsia. Declining incidence in HICs may reduce clinical awareness, while eclampsia remains a major challenge in MICs. International consensus on best practices, strengthened surveillance systems and sustained multinational collaborations/networks such as INOSS, supported by governmental investment, are crucial to improving prevention, management and outcomes worldwide.

## Author Contributions

S.D.: conceptualised the project; D.M., S.S.: curated the data; D.M., H.M.E., R.R.: conducted the formal analysis; S.S., D.M., C.D.‐T., H.M.E., L.R.K., D.K., A.K., K.K., A.L., C.T.M., T.P.S., G.V., K.J.C.V., S.D.: conducted investigations and validated the results; S.D., S.S., D.M.: wrote the original draft of the manuscript. All authors reviewed and edited the manuscript and accepted the final version to be published. All authors have full access to all the data in the study and accepting responsibility for the decision to submit for publication.

## Funding

The authors have nothing to report. National funders had no role in data collection, analysis, interpretation or writing of the report. Details of national funding for the original studies are in the Supporting Information (Table S7).

## Ethics Statement

The multi‐country analysis was approved by the Ethics Committee of the Istituto Superiore di Sanità (Italian National Health Institute, INHI) (Prot. PRE‐544/17, Rome 18/07/2017).

## Consent

In Italy, France, Slovakia, Suriname and the Netherlands, informed consent was not required as data were part of EOSS activities, and in Norway and Belgium, the study was exempt from individual consent.

## Conflicts of Interest

The authors declare no conflicts of interest.

## Supporting information


**Data S1:** bjo70216‐sup‐0001‐Supinfo.docx.

## Data Availability

The data that support the findings of this study are available on request from the corresponding author. The data are not publicly available due to privacy or ethical restrictions.
