# Supplementary material for: Eclampsia Incidence, Management and Outcomes Across Multi‐Country Surveillance Cohorts: Individual Participant Data Meta‐Analysis
Source: BJOG. 2026 Mar 26;133(8):1631–42. doi: 10.1111/1471-0528.70216 (PMC13254014; doi:10.1111/1471-0528.70216)
Supplement: Supplementary file 1 — Data S1: bjo70216‐sup‐0001‐Supinfo.docx. [file BJO-133-1631-s001.docx]

Supplementary file

Supplementary file for article titled “Eclampsia incidence, management, and outcomes across multi-country surveillance cohorts: individual participant data meta-analysis”

**Contents**

Table S1. Eclampsia definitions 1

**Table S2.** Distribution of findings in eclampsia across the adopted criteria by country 2

**Table S3.** Maternal and perinatal outcomes - year 2019 3

**Table S4.** Descriptive characteristics of the reference studies 4

[Table S5. List of variables 5](#_TOC_250036)

**Table S6.** Maternal characteristics and source population 6

**Table S7**. National funding source details 8

Supporting Figures

[**Figure S1** Incidence of eclampsia across HICs 9](#_TOC_250035)

[**Figure S2** Diagnosis of preeclampsia across all participating countries 9](#_TOC_250034)

**Figure S3**. Premonitory symptoms of eclampsia: pooled proportion of headache 10

[**Figure S4**. Premonitory symptoms of eclampsia: pooled proportion of epigastric pain 10](#_TOC_250033)

[**Figure S5**. Diagnostic criteria of eclampsia: pooled proportion of hypertension (systolic > 140 mmHg and/or diastolic > 90 mmHg) 11](#_TOC_250032)

**Figure S6**. Diagnostic criteria of eclampsia: pooled proportion of thrombocytopenia (platelet

count < 100x109/L) 11

**Figure S7**. Diagnostic criteria of eclampsia: pooled proportion of increased ALT or AST

(> double) 12

[**Figure S8a**. Timing of first fit: pooled proportion of antepartum cases 12](#_TOC_250031)

[**Figure S8b**. Timing of first fit: pooled proportion of intrapartum cases 13](#_TOC_250030)

[**Figure S8c**. Timing of first fit: pooled proportion of postpartum cases 13](#_TOC_250029)

[**Figure S9**. Number of fits: pooled proportion of cases with one fit 14](#_TOC_250028)

[**Figure S9a**. Number of fits: pooled proportion of cases with one fit (restricted to HICs) 14](#_TOC_250027)

[**Figure S10**. Number of fits: pooled proportion of cases with two fits 15](#_TOC_250026)

[**Figure S11**. Number of fits: pooled proportion of cases with >2 fits 15](#_TOC_250025)

[**Figure S11a**. Number of fits: pooled proportion of cases with >2 fits (restricted to HICs) 16](#_TOC_250024)

[**Figure S12**. Location of first fits: pooled proportion of in-hospital cases 16](#_TOC_250023)

[**Figure S13**. Location of first fits: pooled proportion of at-home cases 17](#_TOC_250022)

[**Figure S14**. Eclampsia treatment: pooled proportion of MgSO4 administration 17](#_TOC_250021)

[**Figure S15**. Eclampsia treatment: pooled proportion of antihypertensive drugs 18](#_TOC_250020)

[**Figure S16**. Eclampsia treatment: pooled proportion of other anticonvulsants 18](#_TOC_250019)

[**Figure S17**. Mode of delivery: pooled proportion of CSs 19](#_TOC_250018)

[**Figure S17a**. Mode of delivery: pooled proportion of antepartum among CSs 19](#_TOC_250017)

[**Figure S17b**. Mode of delivery: pooled proportion of intrapartum among CSs 20](#_TOC_250016)

[**Figure S17c**. Mode of delivery: pooled proportion of postpartum among CSs 20](#_TOC_250015)

[**Figure S18**. Mode of delivery: pooled proportion of urgent/emergency CSs 21](#_TOC_250014)

[**Figure S18a**. Mode of delivery: pooled proportion of antepartum among urgent/emergency CSs 21](#_TOC_250013)

[**Figure S18b**. Mode of delivery: pooled proportion of intrapartum among urgent/emergency CSs 22](#_TOC_250012)

[**Figure S19**. Maternal outcomes: pooled proportion of preterm deliveries 22](#_TOC_250009)

[**Figure S19a**. Maternal outcomes: pooled proportion of antepartum among preterm deliveries 23](#_TOC_250008)

[**Figure S19b**. Maternal outcomes: pooled proportion of intrapartum among preterm deliveries 23](#_TOC_250007)

[**Figure S19c**. Maternal outcomes: pooled proportion of postpartum among preterm deliveries 24](#_TOC_250006)

[**Figure S20**. Maternal outcomes: pooled proportion of higher-level monitoring admission 24](#_TOC_250011)

[**Figure S20a.** Maternal outcomes: pooled proportion of higher-level monitoring admission (restricted to HICs) 25](#_TOC_250010)

[**Figure S21**. Maternal outcomes: pooled proportion of HELLP syndrome 25](#_TOC_250005)

[**Figure S22**. Maternal outcomes: pooled proportion of PRES (restricted to HICs) 26](#_TOC_250004)

[**Figure S23**. Neonatal outcomes: pooled proportion of Apgar 5th <7 26](#_TOC_250003)

[**Figure S24**. Neonatal outcomes: pooled proportion of NICU admission 27](#_TOC_250002)

[**Figure S24a**. Neonatal outcomes: pooled proportion of NICU admission (restricted to HICs) 27](#_TOC_250001)

[References 28](#_TOC_250000)

| **Eclampsia definitions** | |
| --- | --- |
| **Belgium** | Any woman presenting with convulsion(s) during pregnancy or within the first 10 days after delivery, in combination with at least 2 of the following features within 24 hours of the convulsion(s): hypertension (a maximum diastolic blood pressure of >= 90 mmHg and a diastolic increment of >= 25 mmHg, having had a diastolic blood pressure <90 mmHg at the first antenatal visit) proteinuria (at least + protein in a random urine sample or >= 0.3 g of proteins in a 24-hour collection |
| **France** | Seizures in a woman diagnosed with preeclampsia, and if not, not attributable to another cause during pregnancy and up to 42 days postpartum |
| **Italy** | Any woman with convulsion (s) during pregnancy or in the first 14 days post-partum, without another more likely cause and associated to at least one of the following signs: hypertension (systolic > 140 mmHg and/or diastolic > 90 mmHg); proteinuria (24 h-urine collection with a total protein excretion of >300 mg or as >1 g/l in a random urine sample or > ‘2+’ on urine dipstick or as a protein/creatinine ratio >0.3 mg/ ml [30 mg/mmol]); thrombocytopenia (platelet count of less than 100x109/L); raised plasma alanine aminotransferase concentration (ALT)  or an increased plasma aspartate aminotransferase (AST) concentration (more than double in comparison to normal values) (INOSS Definition). |
| **Netherlands** | Occurrence of generalized convulsions during pregnancy or within the first 10 days postpartum, not attributable to other causes |
| **Norway** | Generalized convulsions during pregnancy, delivery or the first 7 days postpartum, in a woman with preeclampsia or pregnancy hypertension (any severity), not attributable to other causes. |
| **Slovakia** | Occurrence of generalized convulsions during pregnancy or within the first 10 days postpartum, not attributable to other causes |
| **Suriname** | Any woman with convulsion (s) during pregnancy or in the first 14 days post-partum, without another more likely cause and associated to at least one of the following signs: hypertension (systolic > 140 mmHg and/or diastolic > 90 mmHg); proteinuria (24 h-urine collection with a total protein excretion of >300 mg or as >1 g/l in a random urine sample or > ‘2+’ on urine dipstick or as a protein/creatinine ratio >0.3 mg/ ml [30 mg/mmol]); thrombocytopenia (platelet count of less than 100x109/L); raised plasma alanine aminotransferase concentration (ALT)  or an increased plasma aspartate aminotransferase (AST) concentration (more than double in comparison to normal values) (INOSS Definition). |
| **Table S1. Eclampsia definitions^1-4^** | |

|  | **BELGIUM** | **FRANCE** | **ITALY** | **NETHERLANDS** | **NORWAY** | **SLOVAKIA** | **SURINAME** | **Pooled estimates** | | |
| --- | --- | --- | --- | --- | --- | --- | --- | --- | --- | --- |
|  | n=64 (%) | n=51 (%) | n=109 (%) | n=88 (%) | n=174 (%) | n=57 (%) | n=72 (%) | % (95% CI) | | |
| **Hypertension** |  |  |  |  |  |  |  |  | | |
| Yes | 55 (87.3%) | 43 (84.3%) | 98 (90.7%) | 43 | NA | 54 (98.2%) | 65 (94.2%) | 91.1 (73.0-94.9) | | |
| No | 8 (12.7%) | 8 (15.7%) | 10 (9.3%) | 6 | NA | 1 (1.8%) | 4 (5.8%) |  | | |
| *Missing* | *1 (1.6%)* | *0 (0.0%)* | *1 (0.9%)* | *39§* |  | *2 (3.5%)* | *3 (4.2%)* |  | | |
| **Proteinuria** |  |  |  |  |  |  |  |  | | |
| Yes | 35 | 32 (64.0%) | 46 (42.2%) | 38 | NA | 31 | 37 | - | | |
| No | 9 | 18 (36.0%) | 63 (57.8%) | 5 | NA | 4 | 11 |  | | |
| *Missing* | *20§* | *1 (2.0%)* | *0 (0.0%)* | *45§* |  | *22§* | *24§* |  | | |
| **Thrombocytopenia** |  |  |  |  |  |  |  |  | | |
| Yes | 10 (16.4%) | 19 (38.8%) | 38 (34.9%) | 12 | NA | 17 (32.7%) | 10 (14.5%) | 27.3 (17.0-42.3) | | |
| No | 51 (83.6%) | 30 (61.2%) | 71 (65.1%) | 45 | NA | 35 (67.3%) | 59 (85.5%) |  | | |
| *Missing* | *3 (4.7%)* | *2 (3.9%)* | *0 (0.0%)* | *31§* |  | *5 (8.8%)* | *3 (4.2%)* |  | | |
| **Increased plasma AST or ALT** | | | | | | | | | | |
| Yes | 34 (55.7%) | 20 (46.5%) | 29 (26.6%) | 19 | NA | 18 (34.6%) | 13 (19.7%) | 35.7 (22.3-52.8) | | |
| No | 27 (44.3%) | 23 (53.5%) | 80 (73.4%) | 36 | NA | 34 (65.4%) | 53 (80.3%) |  | | |
| *Missing* | *3 (4.7%)* | *8 (15.7%)* | *0 (0.0%)* | *33§* |  | *5 (8.8%)* | *6 (8.3%)* |  | |  |
| Data: n (%). §Variable excluded due to >25% missing data. NA=not available. ALT=alanine aminotransferase. AST= aspartate aminotransferase  Hypertension: systolic > 140 mmHg and/or diastolic > 90 mmHg; proteinuria: 24 h-urine collection with total protein excretion of >300 mg or as >1 g/l in a random urine sample or > ‘2+’ on urine dipstick or protein/creatinine ratio >0.3 mg/ ml [30 mg/mmol]); thrombocytopenia: platelet count <100x10^9^/L; increased plasma AST or ALT: > double in comparison to normal values. | | | | | | | | |  |  |
| **Table S2. Distribution of findings across the adopted criteria by country** | | | | | | | | |  |  |

|  | **Belgium** | **France** | **Italy** | **Netherlands** | **Norway** | **Slovakia** | **Suriname*** |
| --- | --- | --- | --- | --- | --- | --- | --- |
| **Maternal age at delivery >35 years^#^** | 20.6% | 23.0% | 34.4% | 22.4% | 21.8% | 18.5% | 11.4% |
| **Stillbirths (≥28 weeks)** | 3.2‰ | 2.8‰ | 2.2‰ | 2.3‰ | 2‰ | 3.5‰ | 15.6‰ |
| **Preterm deliveries^¶^** | 8.1% | 6.9% | 7.5% | 6.5% | 6.1% | 7.2% | 14.0% |
| **Caesarean Sections^‡^** | 21.5% | 20.9% | 33.0% | 17.4% | 16.4% | 30.1% | 24.1% |
| **MMR (95% CI)**** | 11.4 (8.1-15.6) | 8.0 (7.0-9.3) | 8.7 (7.2-10.5) | 4.7 (3.3-6.4) | 2.7 (1.2-5.4) | 10.9 (7.4-15.5) | 112 (NA) |
| **HDP specific MMR (95% CI)**** | 0.9 (0.2-2.6) | 0.2 (0.0-0.4) | 0.9 (0.4-1.5) | 0.7 (0.2-1.5) | 0 (0.0-1.3) | 1.1 (0.2-3.1) | 33.3 (NA) |
| * Years: 2016-17.  # Percentages on live and stillbirths.  ¶ Percentages on live births.  ‡ Percentages on total births.  ** MMR: France and Italy: 2013-15; the Netherlands, Norway and Slovakia: 2014-18; Belgium: 2021-2023. MMR=maternal mortality ratio/100.000 live births; HDP= hypertensive disorders of pregnancy; | | | |  |  |  |  |
| **Table S3. Maternal and perinatal outcomes-year 2019^5-7^** | | | | | | | |

|  | **Study period** | **Data collection** | **Source population size (national coverage)** | **Source population source** | **EOSS**  **Availability** | **Audits/ confidential enquiries** |
| --- | --- | --- | --- | --- | --- | --- |
| **Belgium** | 2012-14 | Prospective | 370.925 (98.6%) | Perinatal Register | yes, since 2012 | no |
| **France** | 2012-13 | Prospective | 182,309 (20%) | Birth registers from maternity units | yes, since 2012 | yes |
| **Italy** | 2017-19 | Prospective | 741,516 (75%) | National Health Discharge database | yes, since 2013 | yes |
| **Netherlands** | 2013-16 | Prospective | 502,559 (97%) | NA | yes, since 2013 | yes |
| **Norway** | 2012-19 | Prospective | 462,448 (100%) | Medical Birth Register | NA | no |
| **Slovakia** | 2012-18 | Retrospective | 332,174 (85%) | National Case Register | NA | only audits |
| **Suriname** | 2017-19 | Prospective | 19,652 (90%) | National vital statistics | yes, since 2017 | yes |
| NA=not available | | | | | | |
| **Table S4. Descriptive characteristics of the reference studies^1,2,^ ^8-12^** | | | | | | |

# Table S5. List of variables

| **Maternal demographic information and antenatal characteristics included** |
| --- |
| - Maternal age (<20, 20-39, ≥40 years); |
| - Citizenship or country of birth outside resident country was recorded based on available information (citizen/non-citizen or native/born outside the country of residence); |
| - Educational level stratified as low (≤ primary), intermediate (secondary and professional education), high (university); |
| - Parity (nulliparous/multiparous); |
| - Mode of conception (natural/assisted reproductive technology (ART)); |
| - Multiple pregnancy (yes/no) |
| Diagnostic features |
| - Hypertension (systolic > 140 mmHg and/or diastolic > 90 mmHg) |
| - Proteinuria (24 h-urine collection with a total protein excretion of >300 mg or as >1 g/l in a random urine sample or > ‘2+’ on urine dipstick or protein/creatinine ratio >0.3 mg/ ml [30 mg/mmol]) |
| - Thrombocytopenia (platelet count <100x109/L) |
| - Raised plasma alanine aminotransferase concentration (ALT) or an increased plasma aspartate aminotransferase (AST) concentration (> double in comparison to normal values) |
| Clinical information and fit characteristics |
| - Premonitory symptoms (headache, epigastric pain, nausea/vomiting); |
| - Systolic and diastolic blood pressure values (within 24 hours before and at the fit); |
| - Timing of fit (antepartum, intrapartum, postpartum); |
| - Number of fits (1, 2, ≥ 3); |
| - Location of first fit (hospital, home, other); |
| - Gestational age at fit and at delivery; |
| - Fit-delivery interval categorized by fit timing. |
| Management practices |
| - MgSO4 prophylaxis in women with preeclampsia; |
| - Antihypertensives treatment; |
| - Eclampsia treatment: MgSO4 and other anticonvulsants. |
| Mode of delivery |
| - Cesarean Sections (CSs), stratified by fit timing; |
| - Urgent/Emergency CSs, stratified by fit timing. |
| Maternal outcomes |
| - Preterm deliveries (<37 weeks of gestation), stratified by timing of fit; |
| - Maternal admission to intensive care units (ICU); |
| - Haemolysis, Elevated Liver enzyme levels, and Low Platelet levels (HELLP) syndrome; |
| - Posterior Reversible Encephalopathy Syndrome (PRES); |
| - Maternal deaths during pregnancy, childbirth or ≤42 days after pregnancy ending. |
| Neonatal outcomes |
| - Birthweight; |
| - Apgar 5’ score (<7); |
| - Neonatal ICU (NICU) admission; |
| - Intrauterine foetal deaths (>20 weeks); |
| - Neonatal deaths (<8 days). |

|  | **BELGIUM**  (n=64) | **Source population** (n=376,471)  ***** | **FRANCE**  (n=51) | **Source population** (n=3,650)****** | **ITALY**  (n=109) | **Source population** (n=741,516) | **NETHER LANDS** (n=88) | **Source population** (n=502,559) | **NORWAY**  (n=174) | **Source population** (n=462,448) | **SLOVAKIA**  (n=57) | **Source population** (n=395,339)******* | **SURINAME**  (n=72) | **Source population** (n=18,296)******** |
| --- | --- | --- | --- | --- | --- | --- | --- | --- | --- | --- | --- | --- | --- | --- |
| **Maternal age (years)** |  |  |  |  |  |  |  |  |  |  |  |  |  |  |
| Median | 28 (NA) |  | 24  (21-35) |  | 32  (28-36) |  | 28  (25-34) |  | 28  (25-33) |  | 26  (16 - 41) |  | 23 (NA) |  |
| <20 | 5  (7.8%) | 7566  (2.0%) | 1  (2.0%) | 50  (1.4%) | 6  (5.6%) | NA | 5  (6.4%) | 5262  (1.0%) | 9  (5.2%) | 5457  (1.2%) | 16  (28.1%) | 24469  (6.2%) | 21  (29.2%) | 2454  (13.4%) |
| 20-39 | 58  (90.6%) | 356820  (94.8%) | 48  (94.1%) | 3462  (94.9%) | 88  (81.5%) | NA | 71  (91.0%) | 479193  (95.7%) | 155  (89.1%) | 440300  (95.2%) | 39  (68.4%) | 359878  (91.3%) | 49  (68.1%) | 15212  (83.3%) |
| ≥40 | 1  (1.6%) | 12065  (3.2%) | 2  (3.9%) | 137  (3.8%) | 14  (13.0%) | NA | 2  (2.6%) | 16472  (3.3%) | 10  (5.7%) | 16691  (3.6%) | 2  (3.5%) | 9811  (2.5%) | 2  (2.8%) | 588  (3.2%) |
| *Missing* | *0*  *(0.0%)* | 20  (0.0%) | *0*  *(0.0%)* | *1*  *(0.0%)* | *1*  *(0.9%)* | *NA* | *10*  *(11.4%)* | *1632*  *(0.3%)* | *0*  *(0.0%)* | *0*  *(0.0%)* | *0*  *(0.0%)* | *1181*  *(0.3%)* | *0*  *(0.0%)* | *42*  *(0.2%)* |
| **Citizenship*#*** |  |  |  |  |  |  |  |  |  |  |  |  |  |  |
| Native | NA | - | 29  (58.0%) | 2722  (82.8%) | 72  (69.2%) | 546020  (73.6%) | 45  (68.2%) | 406903  (82.1%) | 120  (70.6%) | 325637  (71.4%) | NA | - | 70  (97.2%) | NA |
| Not native | NA | - | 21  (42.0%) | 565  (17.2%) | 32  (30.8%) | 195496  (26.4%) | 21  (31.8%) | 88776  (17.9%) | 50  (29.4%) | 130487  (28.6%) | NA | - | 2  (2.8%) | NA |
| *Missing* |  |  | *1*  *(2.0%)* | *363*  *(9.9%)* | *5*  *(4.6%)* | *0*  *(0.0%)* | *22*  *(25.0%)* | *6880*  *(1.4%)* | *4*  *(2.3%)* | *6324*  *(1.4%)* |  |  | *0*  *(0.0%)* |  |
| **Educational Level** |  |  |  |  |  |  |  |  |  |  |  |  |  |  |
| Low | NA | - | 5  (12.2%) | NA | 42  (46.2%) | NA | NA | - | 39  (26.9%) | NA | 21  (41.2%) | NA | 15 | - |
| Intermediate | NA | - | 28  (68.3%) | NA | 32  (35.2%) | NA | NA | - | 33  (22.8%) | NA | 19  (37.3%) | NA | 15 | - |
| High | NA | - | 8  (19.5%) | NA | 17  (18.7%) | NA | NA | - | 73  (50.3%) | NA | 11  (21.6%) | NA | 5 | - |
| *Missing* |  |  | *10*  *(19.6%)* |  | *18*  *(16.5%)* |  |  |  | *29*  *(16.7%)* |  | *6*  *(10.5%)* |  | *37§* |  |

*to be continued*

*to be continued*

|  | **BELGIUM (n=64)** | **Source population**  **(n=376,471)** | **FRANCE (n=51)** | **Source population**  **(n=3,650)**** | **ITALY (n=109)** | **Source population**  **(n=741,516)** | **NETHER LANDS**  **(n=88)** | **Source population**  **(n=502,559)** | **NORWAY (n=174)** | **Source population**  **(n=462,448)** | **SLOVAKIA (n=57)** | **Source population**  **(n=395,339)***** | **SURINAME (n=72)** | **Source population**  **(n=18,296)****** |
| --- | --- | --- | --- | --- | --- | --- | --- | --- | --- | --- | --- | --- | --- | --- |
| **Parity** |  |  |  |  |  |  |  |  |  |  |  |  |  |  |
| Multiparous | 18  (28.1%) | 211828  (56.3%) | 25  (49.0%) | 2110  (58.2%) | 40  (36.7%) | 334424  (45.1%) | 19  (23.2%) | 275082  (54.9%) | 48  (27.6%) | 266367  (57.6%) | 14  (24.6%) | 213773  (54.1%) | 26  (36.1%) | 11984  (65.7%) |
| Nulliparous | 46  (71.9%) | 164495  (43.7%) | 26  (51.0%) | 1517  (41.8%) | 69  (63.3%) | 407092  (54.9%) | 63  (76.8%) | 225708  (45.1%) | 126  (72.4%) | 196081  (42.4%) | 43  (75.4%) | 181566  (45.9%) | 46  (63.9%) | 6248  (34.3%) |
| *Missing* | *0*  *(0.0%)* | 148  (0.0%) | *0*  *(0.0%)* | *23*  *(0.6%)* | *0*  *(0.0%)* | *0*  *(0.0%)* | *6*  *(6.8%)* | *1769*  *(0.4%)* | *0*  *(0.0%)* | *0*  *(0.0%)* | *0*  *(0.0%)* | *0*  *(0.0%)* | *0*  *(0.0%)* | *64*  *(0.3%)* |
| **ART** |  |  |  |  |  |  |  |  |  |  |  |  |  |  |
| No | 59  (95.2%) | 351347  (94.5%) | 48  (96.0%) | 3573  (97.9%) | 94  (91.3%) | 718826  (96.9%) | 74  (92.5%) | 323638  (92.7%) | 168  (96.6%) | 446476  (96.5%) | 55  (96.5%) | NA | 72  (100.0%) | 18296  (100%) |
| Yes | 3  (4.8%) | 20600  (5.5%) | 2  (4.0%) | 76  (2.1%) | 9  (8.7%) | 22690  (3.1%) | 6  (7.5%) | 25478  (7.3%) | 6  (3.4%) | 15972  (3.5%) | 2  (3.5%) | NA | 0  (0.0%) | 0  (0.0%) |
| Not known | 2  (3.1%) | 4524  (1.2%) | 1  (2.0%) | 1  (0.0%) | 6  (5.5%) | 0  (0.0%) | 8  (9.1%) | 153443  (30.5%) | 0  (0.0%) | 0  (0.0%) | 0  (0.0%) |  | 0  (0.0%) | 0  (0.0%) |
| **Multiple pregnancy** |  |  |  |  |  |  |  |  |  |  |  |  |  |  |
| No | 61  (96.8%) | 369523  (98.2%) | 47  (92.2%) | 3590  (98.4%) | 101  (92.7%) | 728715  (98.3%) | 81  (98.8%) | 486132  (96.7%) | 163  (93.7%) | 454986  (98.4%) | 54  (94.7%) | 390248  (98.7%) | 71  (98.6%) | 18056  (98.7%) |
| Yes | 2  (3.2%) | 6948  (1.8%) | 4  (7.8%) | 59  (1.6%) | 8  (7.3%) | 12801  (1.7%) | 1  (1.2%) | 16427  (3.3%) | 11  (6.3%) | 7462  (1.6%) | 3  (5.3%) | 5091  (1.3%) | 1  (1.4%) | 240  (1.3%) |
| *Missing* | *1*  *(1.6%)* | *0*  *(0.0%)* | *0*  *(0.0%)* | *1*  *(0.0%)* | *0*  *(0.0%)* | *0*  *(0.0%)* | *6*  *(6.8%)* | *0*  *(0.0%)* | *0*  *(0.0%)* | *0*  *(0.0%)* | *0*  *(0.0%)* | *0*  *(0.0%)* | *0*  *(0.0%)* | *0*  *(0.0%)* |
| **#Norway:** data is referred to the country of birth; The Netherlands: the data is referred to the ethnicity  * **Belgium**: data from Perinatal Registries, excluding three maternity units not participating  ** **France**: randomly selected sample of women without severe maternal morbidity (2% of the total source population)  *** **Slovakia**: data from National Statistics  **** **Suriname**: data from Hospital Registries Data: n (%), median (IQR)  ART=assisted reproductive technologies. NA=not available. §variable excluded due to >25% missing data. | | | | | | | | | | | | | | |
| **Table S6. Maternal characteristics and source population** | | | | | | | | | | | | | | |

| **National funding details** | |
| --- | --- |
| **Belgium** | Belgian Federal Public Service Health. GV was funded by the Flemish Research Foundation (FWO) 2014-2016. |
| **France** | National Research Agency (ANR) |
| **Italy** | Italian Ministry of Health |
| **Netherlands** | NA |
| **Norway** | Norwegian Research Council (Grant n° 320181) |
| **Slovakia** | No funding |
| **Suriname** | No funding |
| **Table S7. National funding source details** | |

# Figure S1. Incidence of eclampsia across high-income countries (HICs)

**
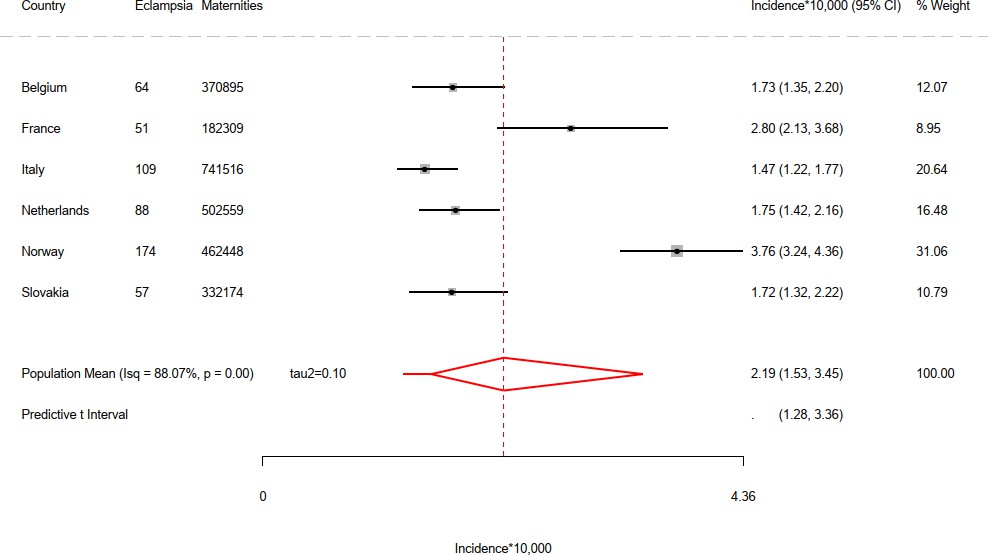
**

# Figure S2. Diagnosis of preeclampsia across all participating countries

**
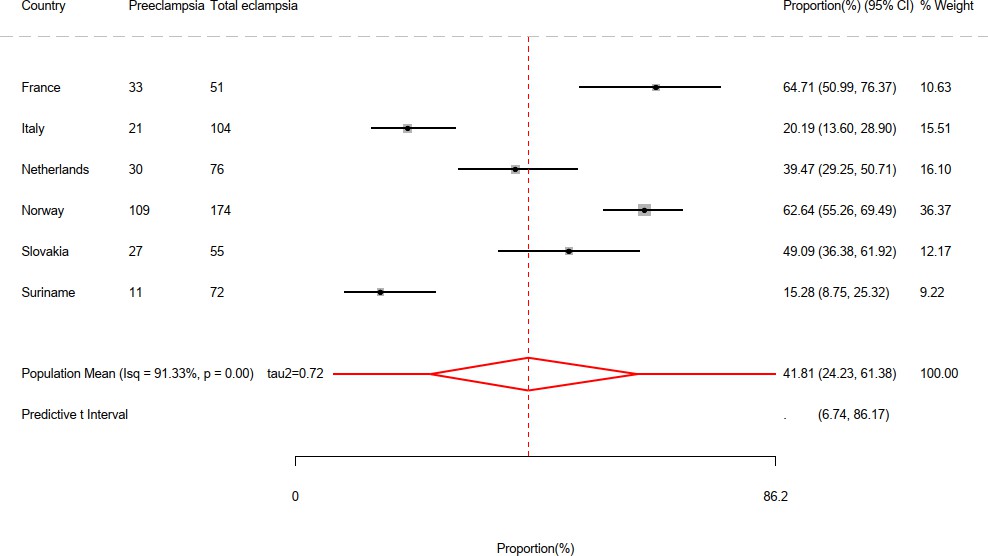
**

**Figure S3. Premonitory symptoms of eclampsia: pooled proportion of headache**

**
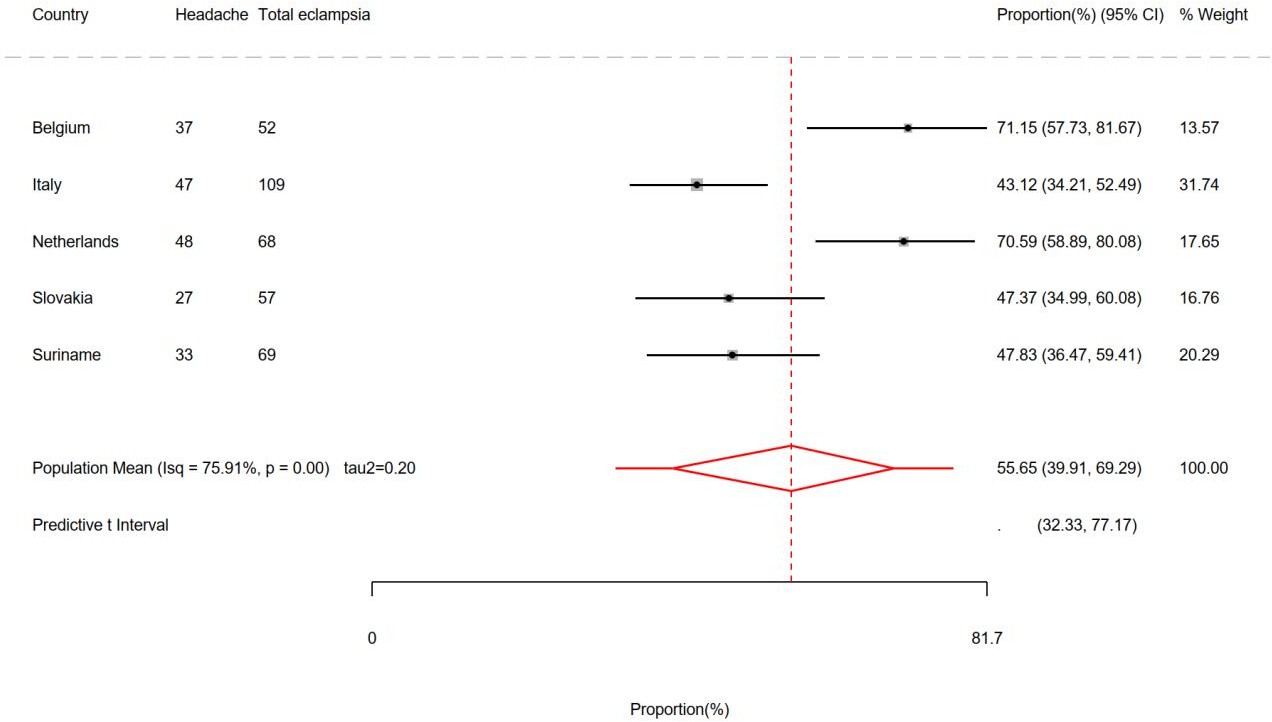
**

# Figure S4. Premonitory symptoms of eclampsia: pooled proportion of epigastric pain

**
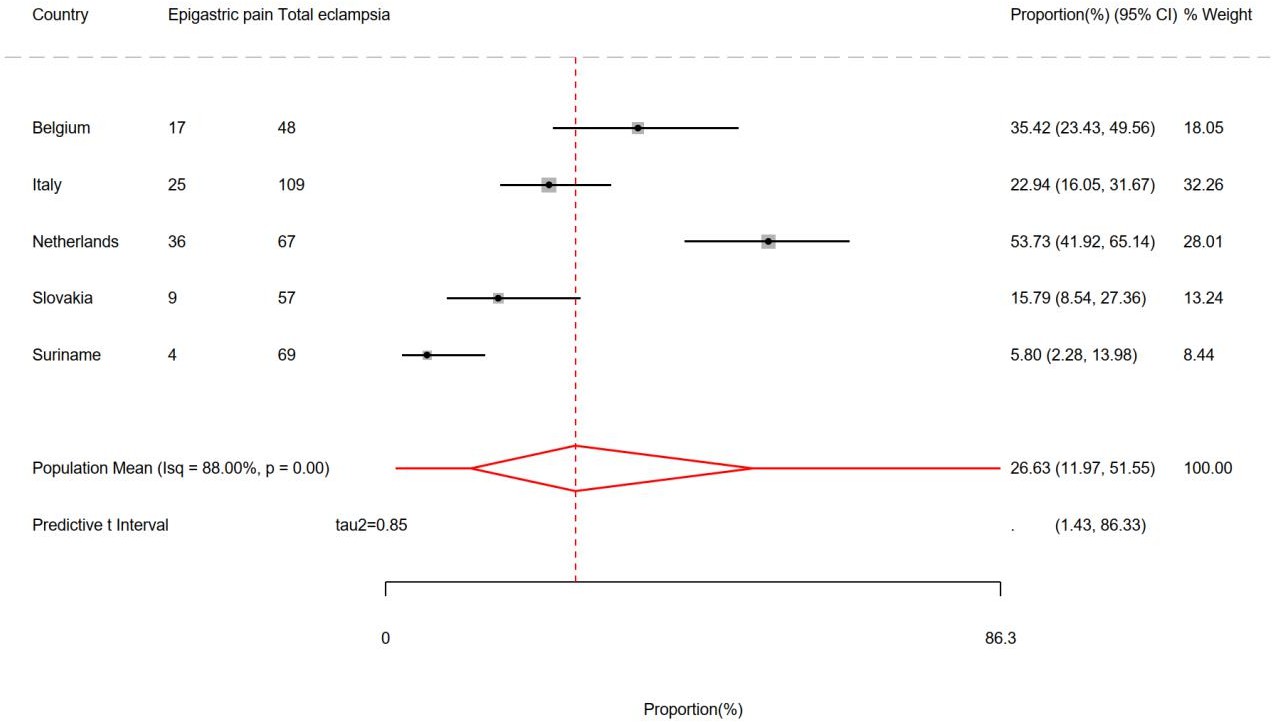
**

# Figure S5. Diagnostic criteria of eclampsia: pooled proportion of hypertension (systolic > 140 mmHg and/or diastolic > 90 mmHg)

**
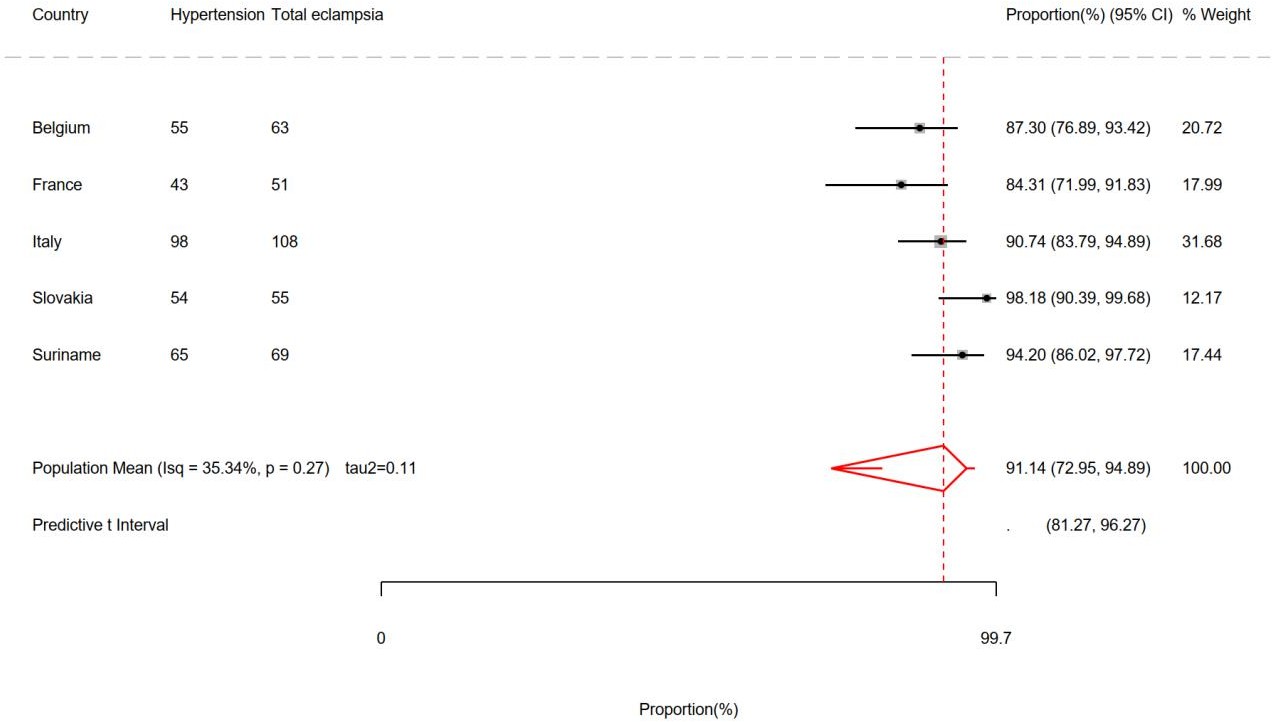
**

**Figure S6. Diagnostic criteria of eclampsia: pooled proportion of thrombocytopenia (platelet count < 100x109/L)**

**
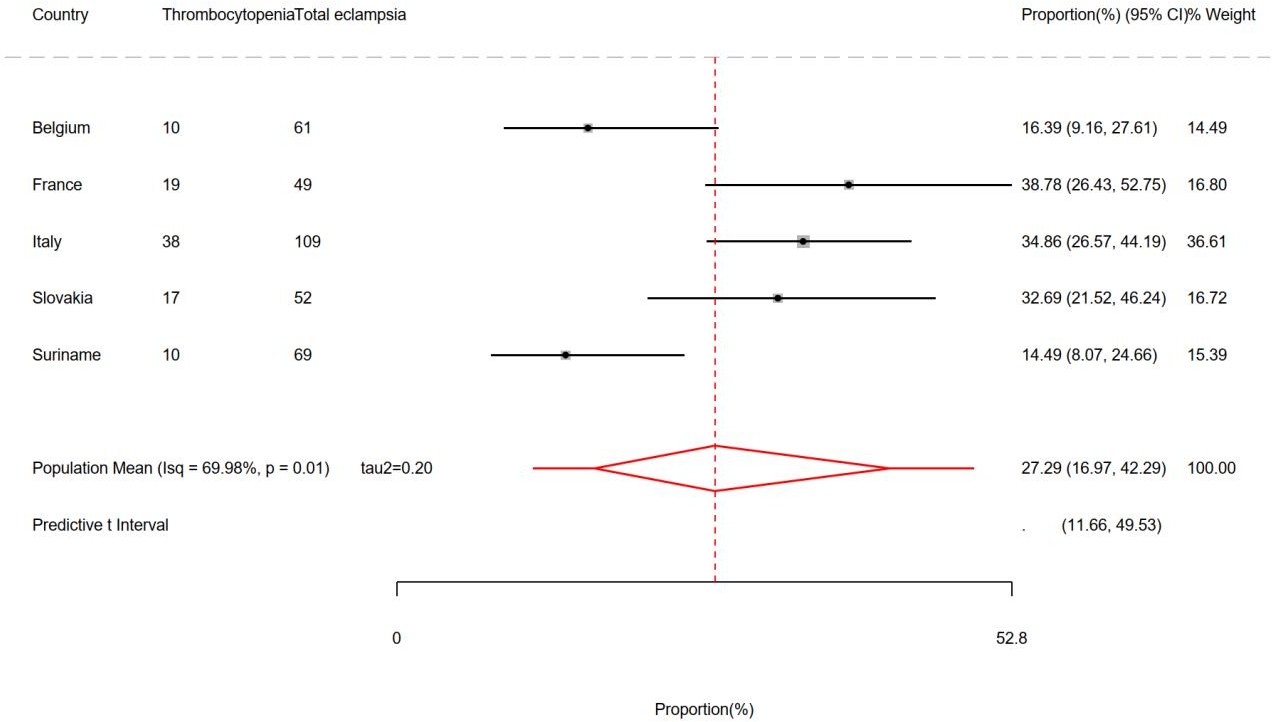
**

**Figure S7. Diagnostic criteria of eclampsia: pooled proportion of increased ALT or AST (> double)**

**
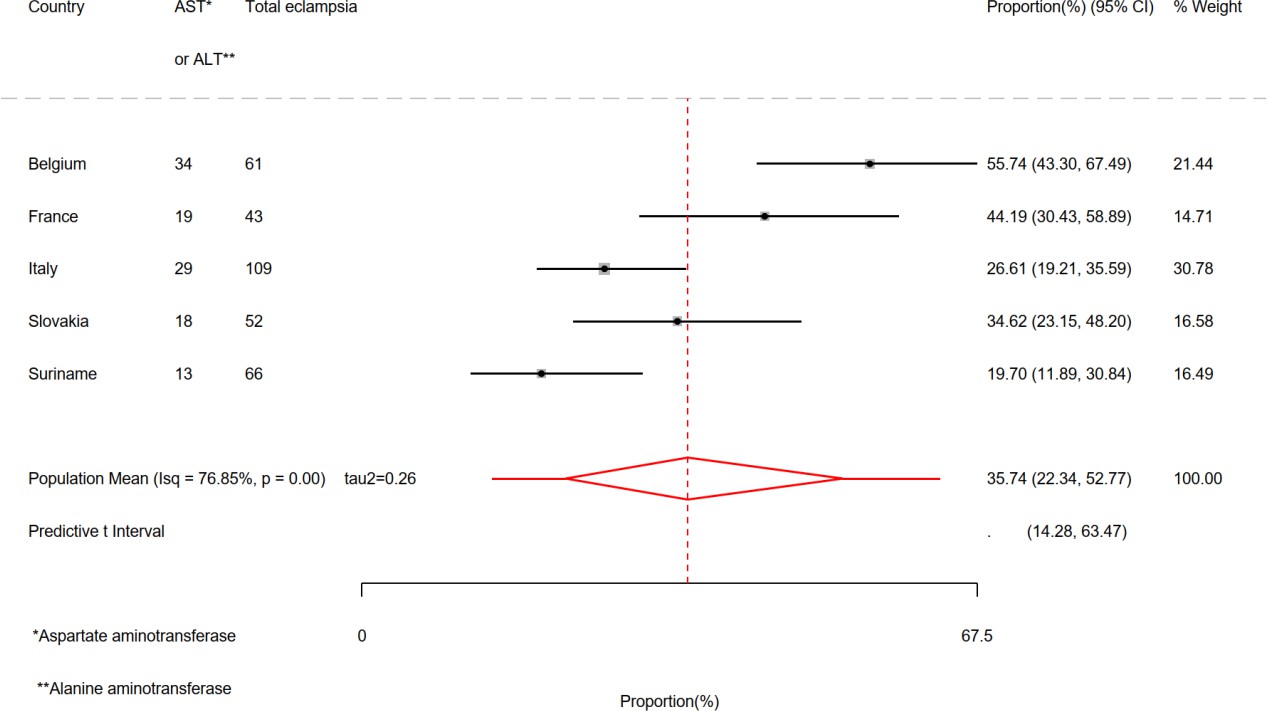
**

# Figure S8a. Timing of first fit: pooled proportion of antepartum cases

**
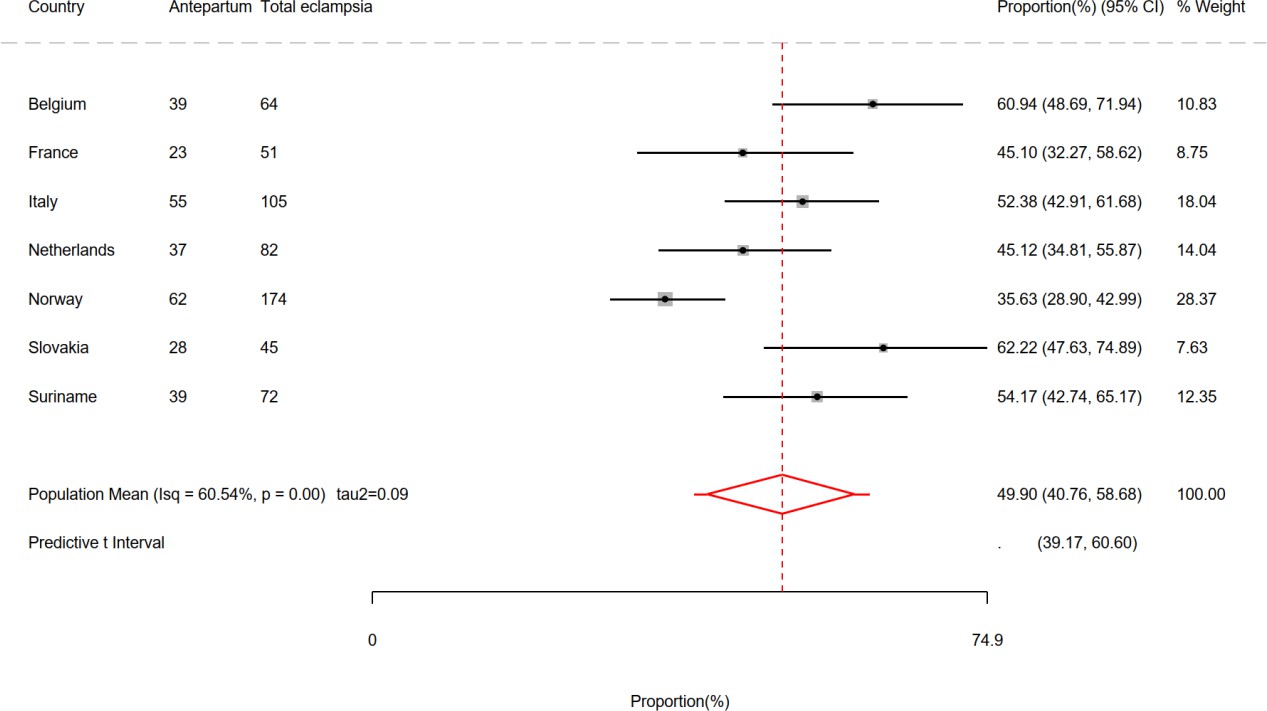
**

# Figure S8b.Timing of first fit: pooled proportion of intrapartum cases

**
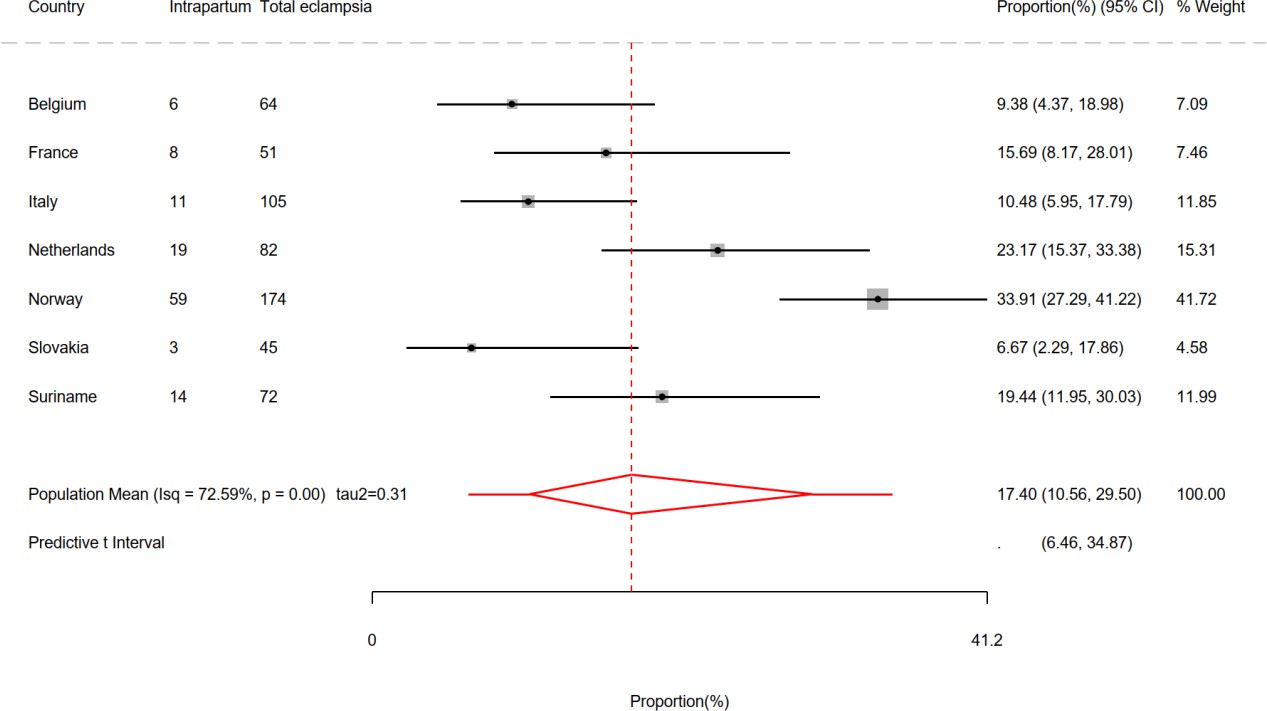
**

# Figure S8c. Timing of first fit: pooled proportion of postpartum cases

**
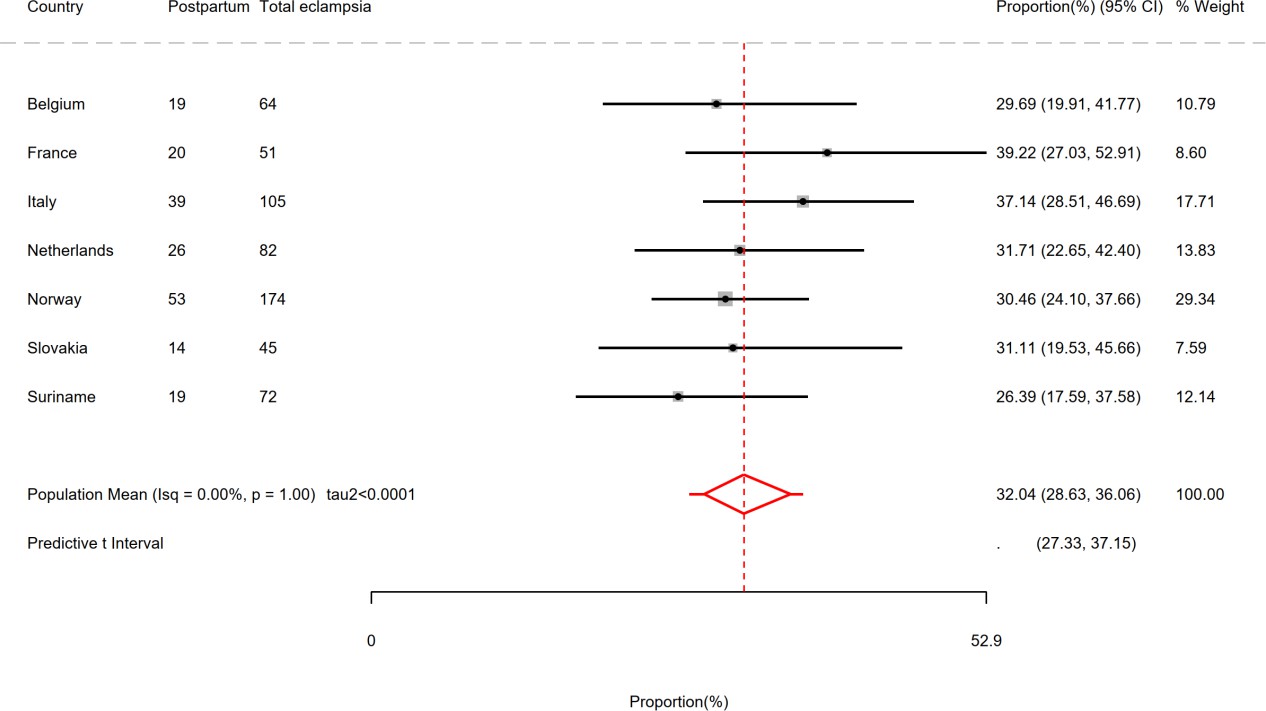
**

# Figure S9. Number of fits: pooled proportion of cases with one fit

**
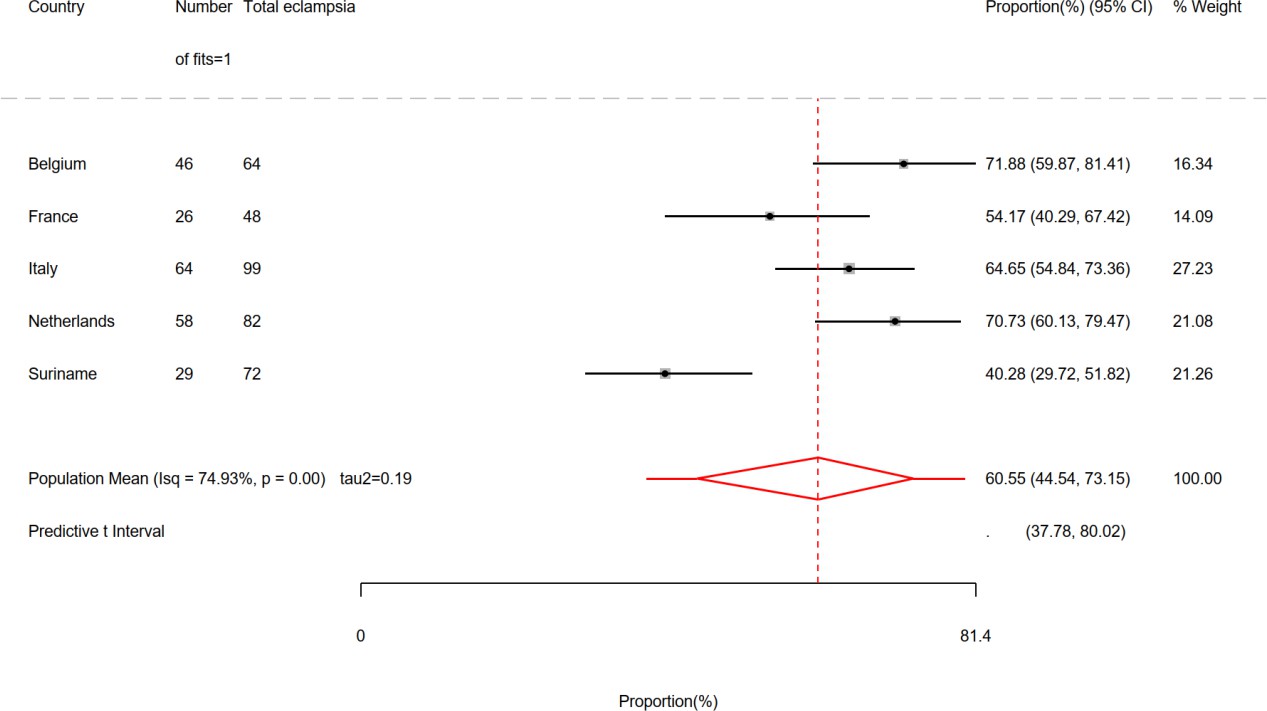
**

# Figure S9a. Number of fits: pooled proportion of cases with one fit (restricted to HICs)

**
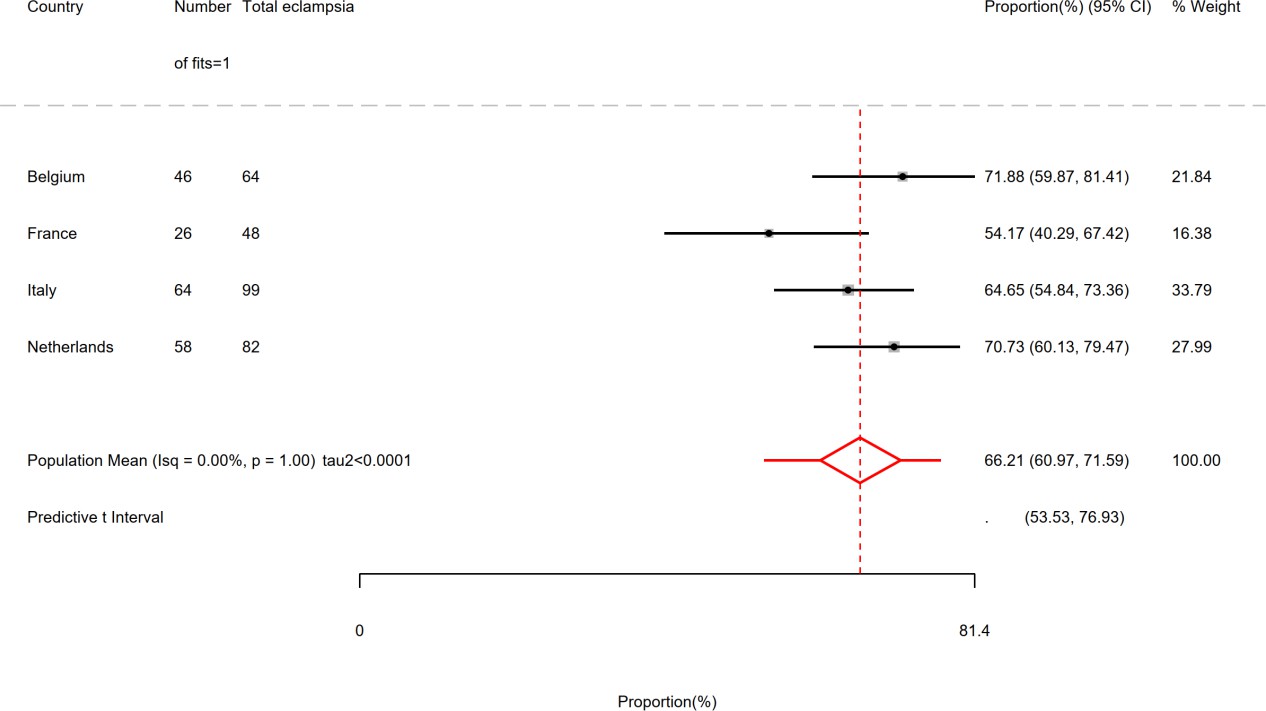
**

# Figure S10. Number of fits: pooled proportion of cases with two fits

**
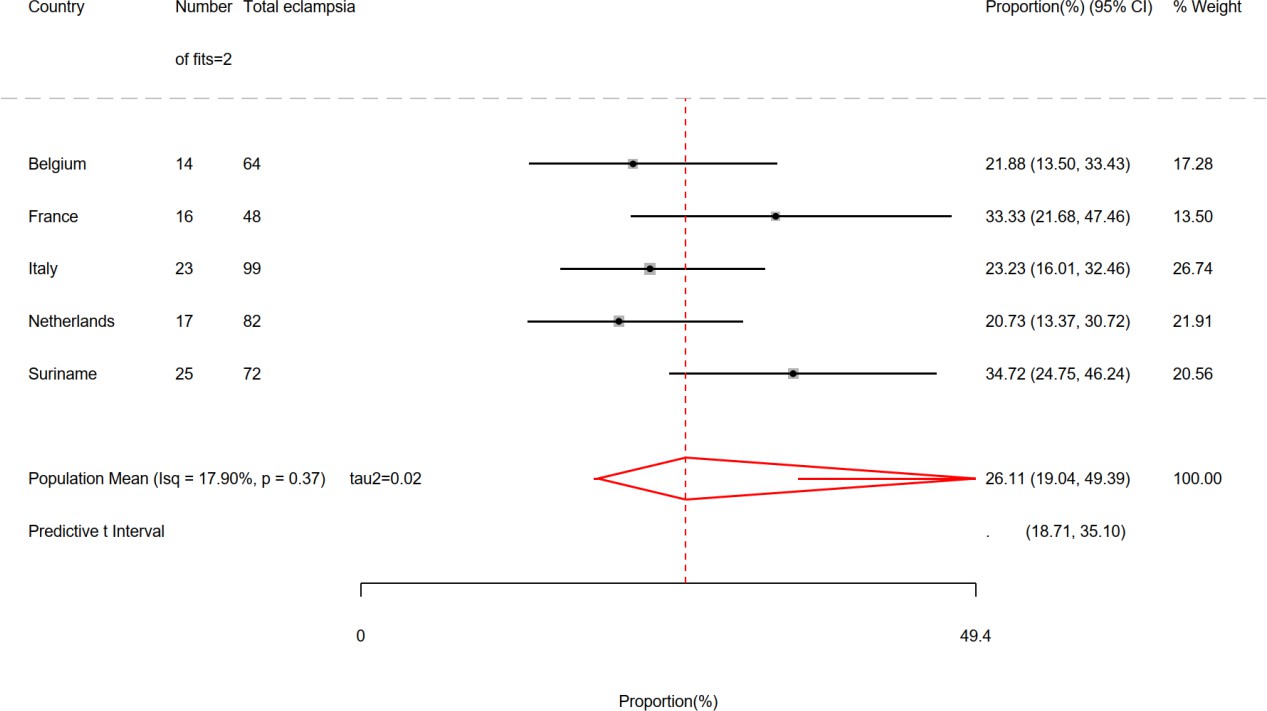
**

# Figure S11. Number of fits: pooled proportion of cases with >2 fits

**
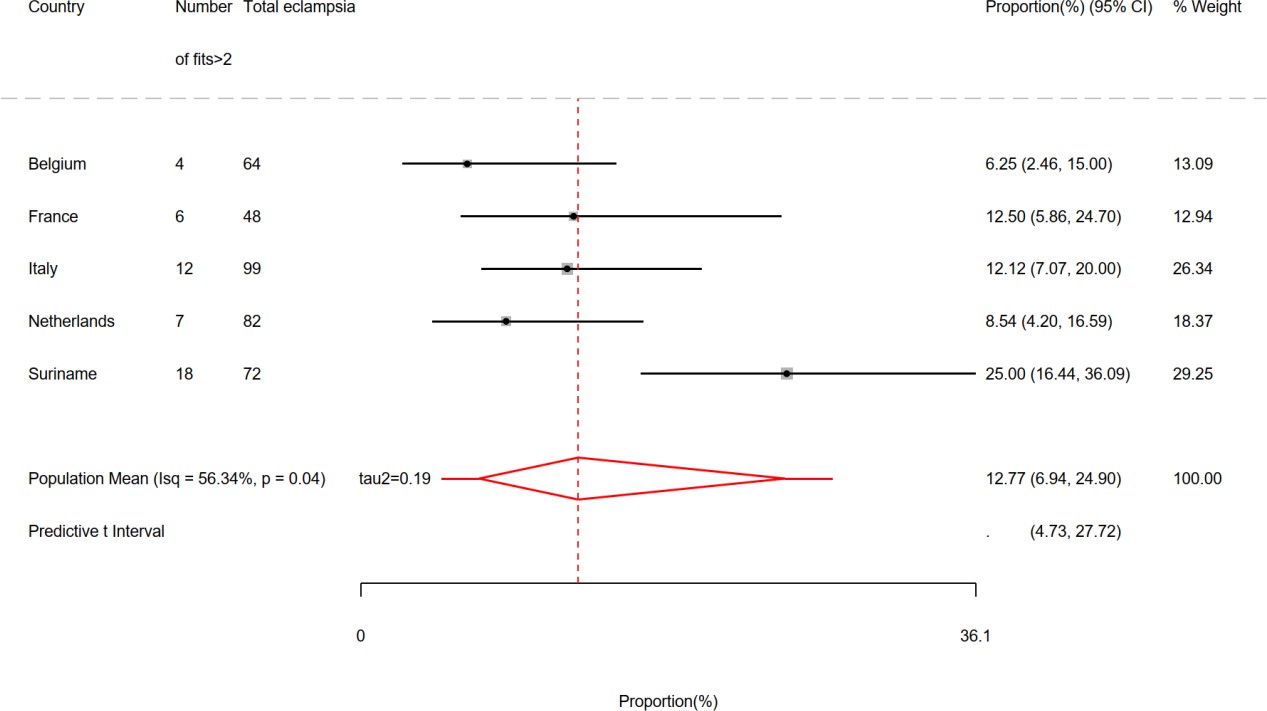
**

# Figure S11a. Number of fits: pooled proportion of cases with >2 fits (restricted to HICs)

**
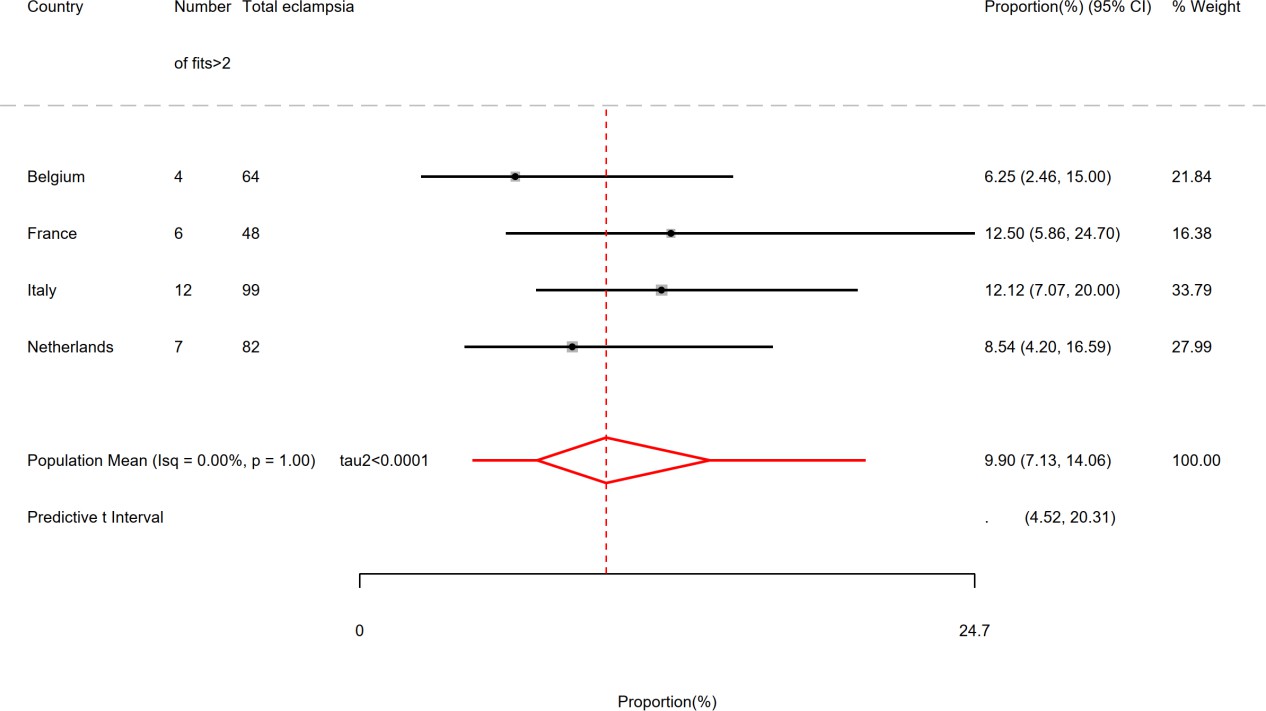
**

# Figure S12. Location of first fits: pooled proportion of in-hospital cases

**
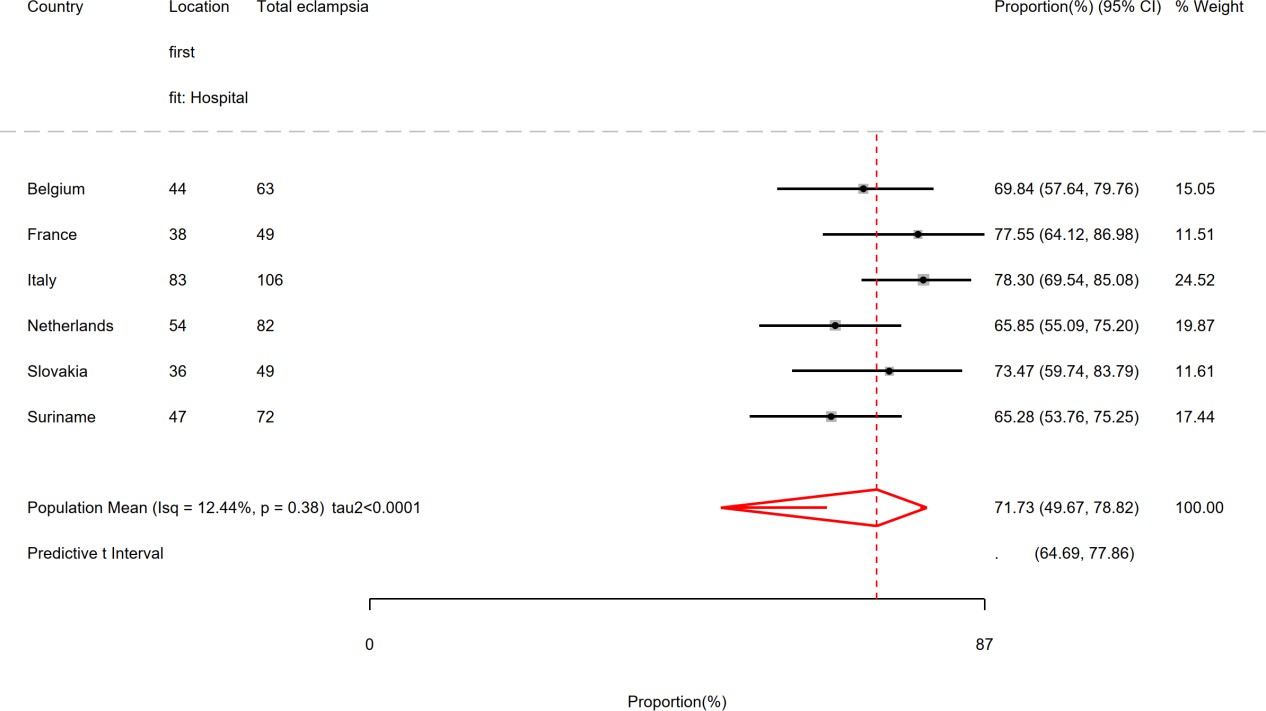
**

# Figure S13. Location of first fits: pooled proportion of at-home cases

**
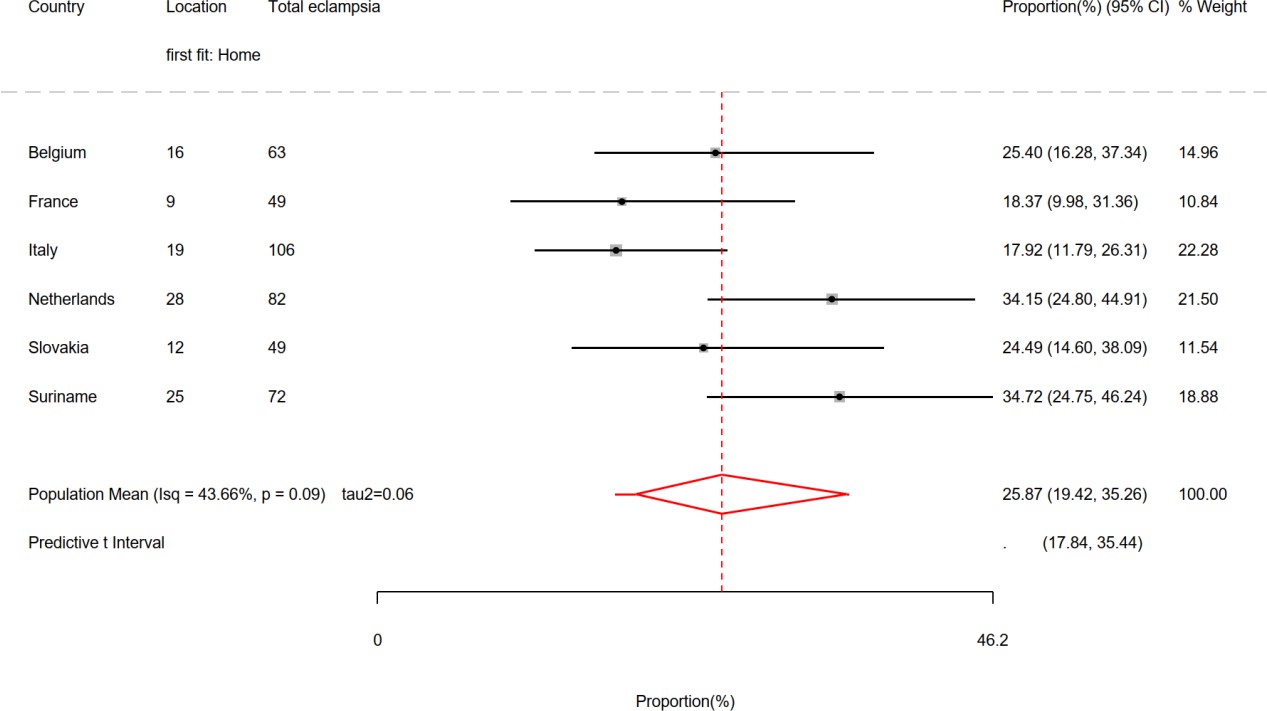
**

# Figure S14. Eclampsia treatment: pooled proportion of MgSO4 administration

**
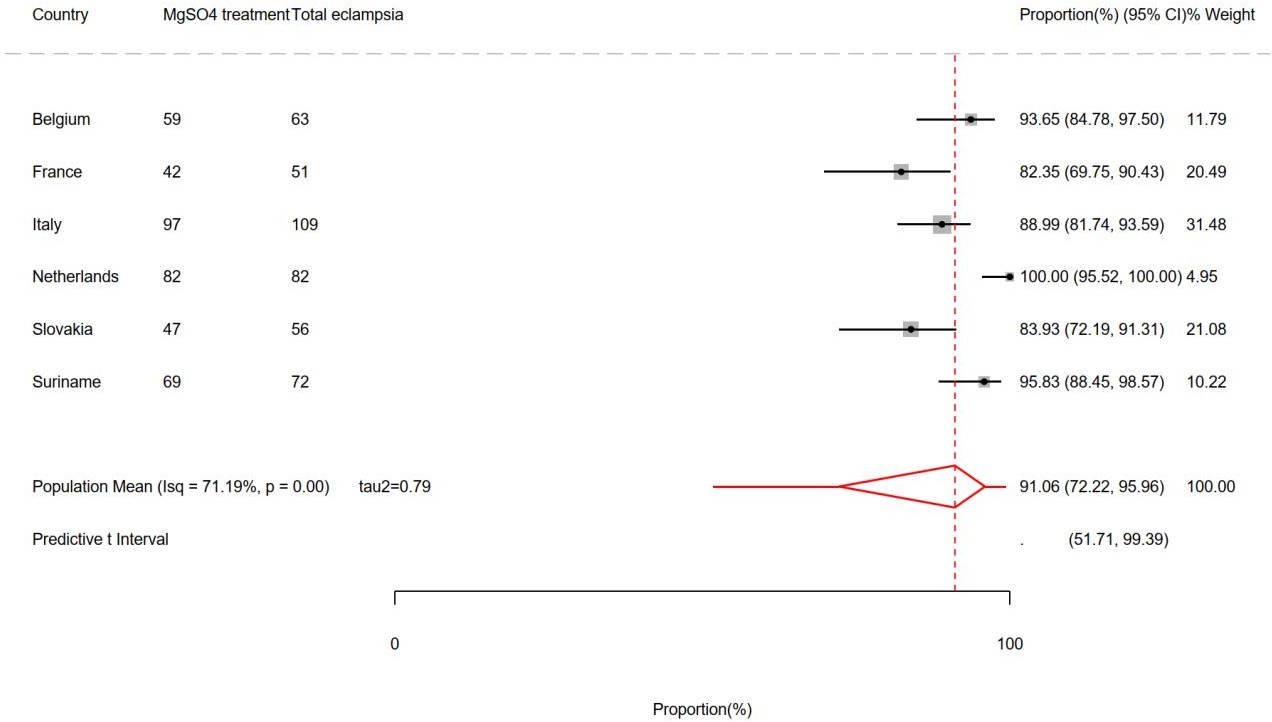
**

# Figure S15. Eclampsia treatment: pooled proportion of antihypertensive drugs

**
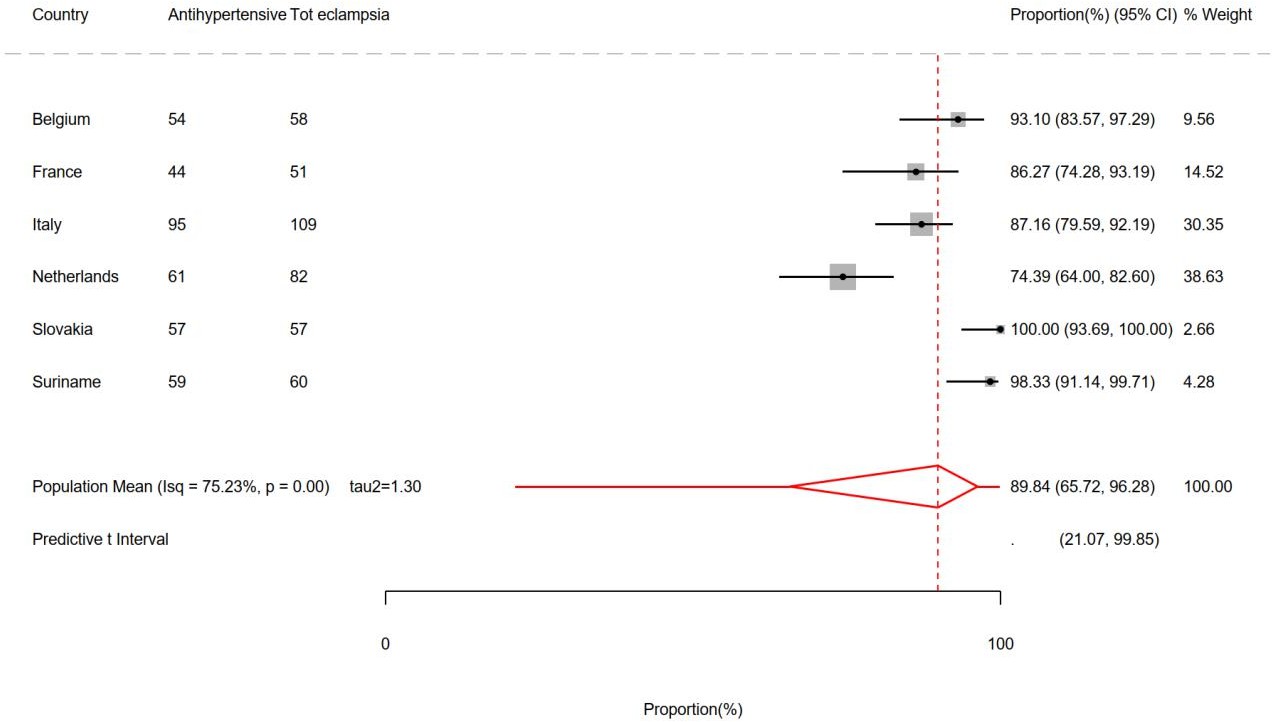
**

# Figure S16. Eclampsia treatment: pooled proportion of other anticonvulsants

**
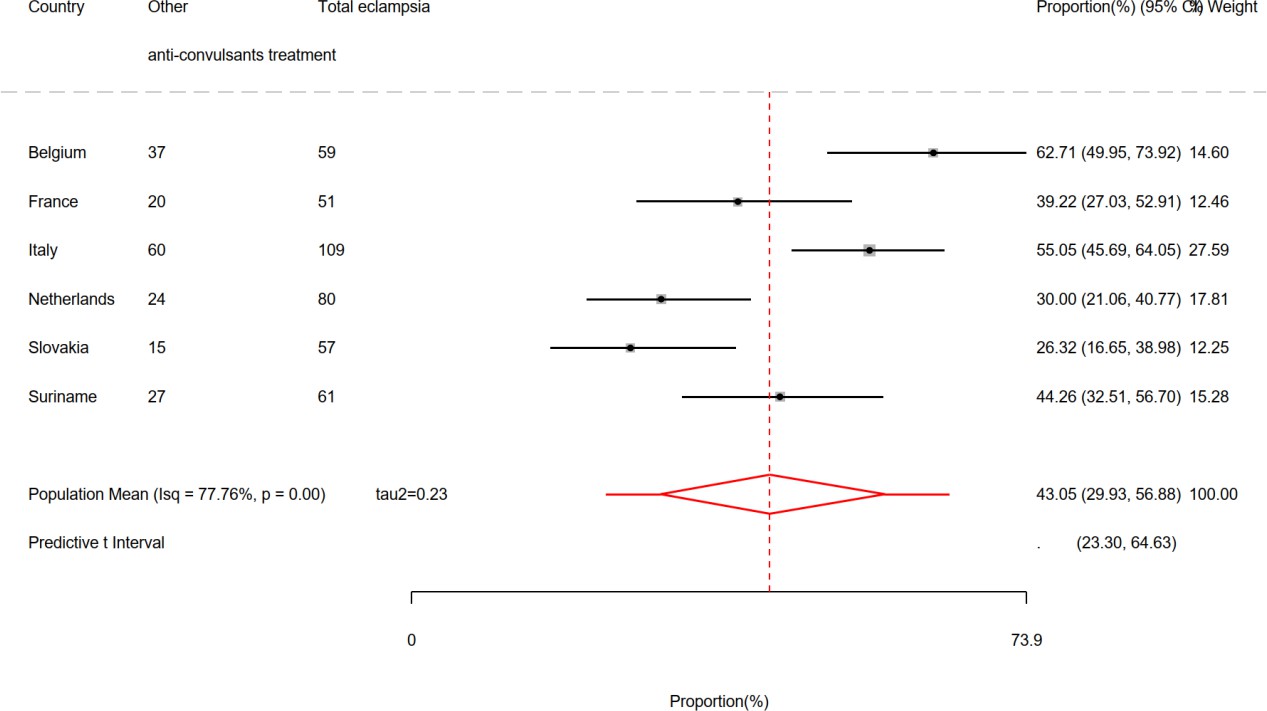
**

# Figure S17. Mode of delivery: pooled proportion of CSs

**
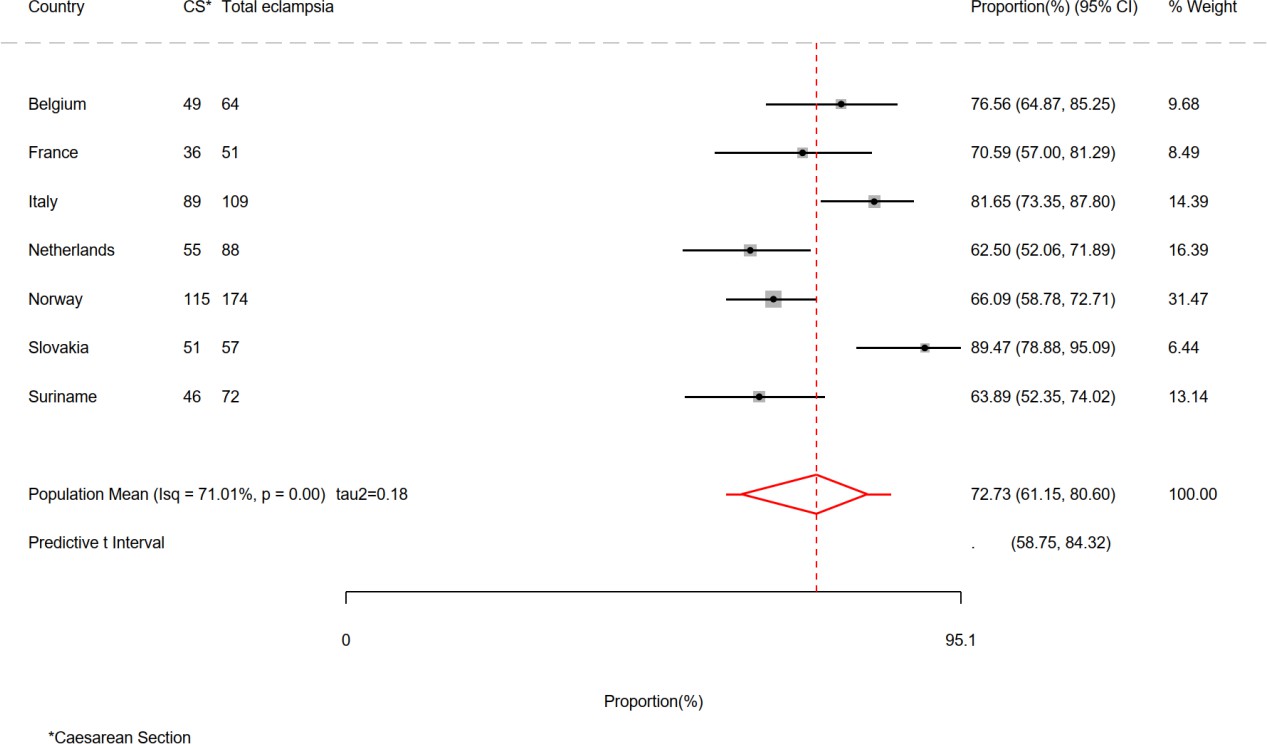
**

# Figure S17a. Mode of delivery: pooled proportion of antepartum among CSs
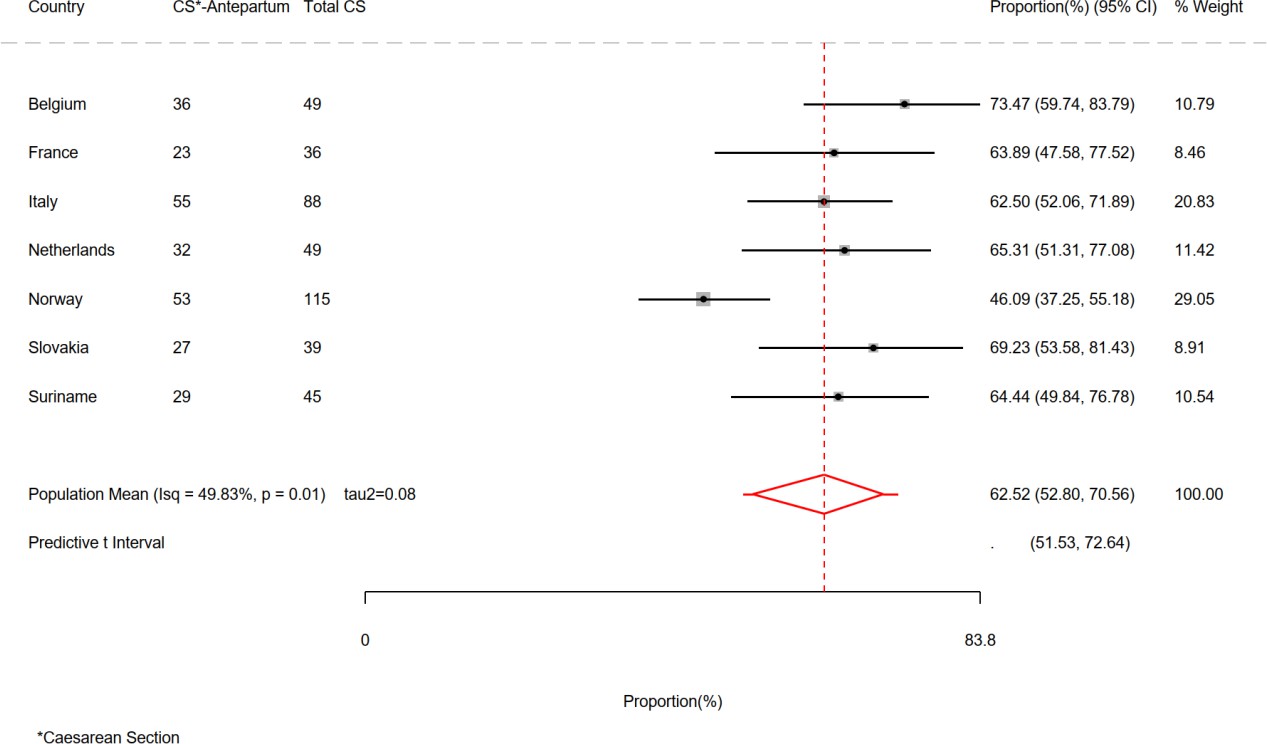


# Figure S17b. Mode of delivery: pooled proportion of intrapartum among CSs
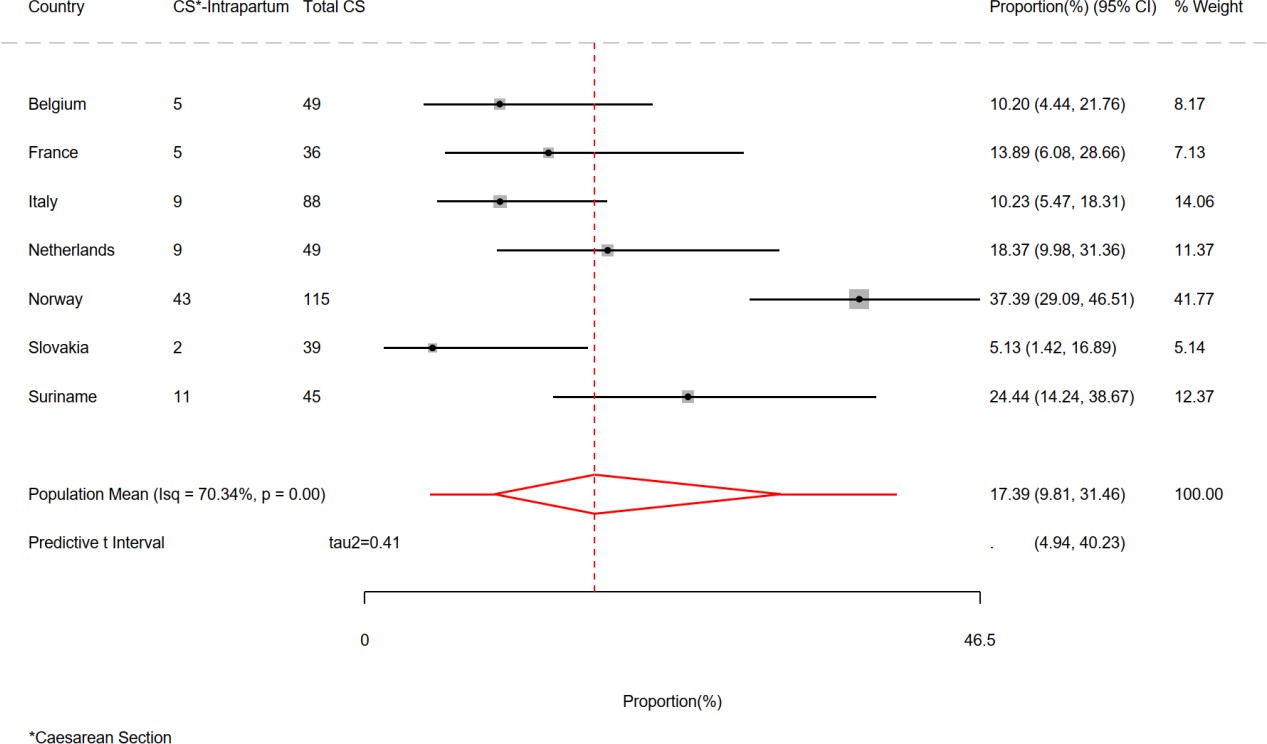


# Figure S17c. Mode of delivery: pooled proportion of postpartum among CSs

**
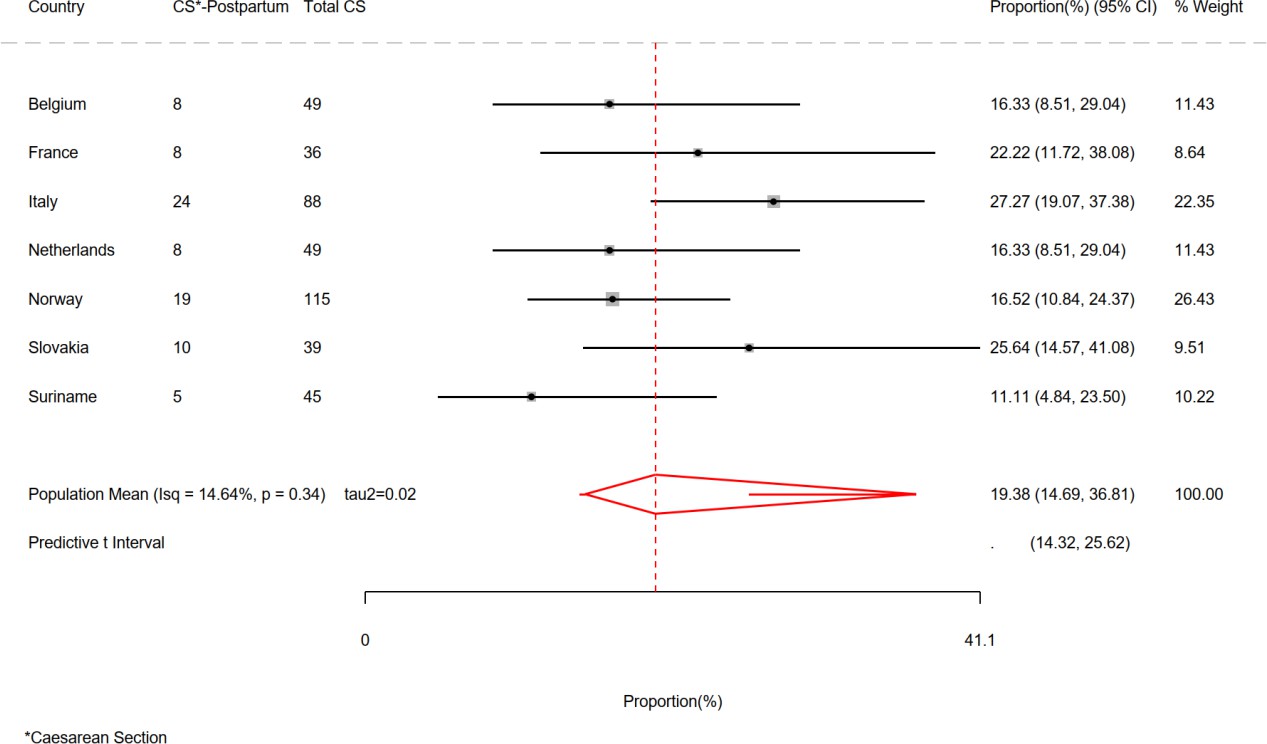
**

# Figure S18. Mode of delivery: pooled proportion of urgent/emergency CSs

**
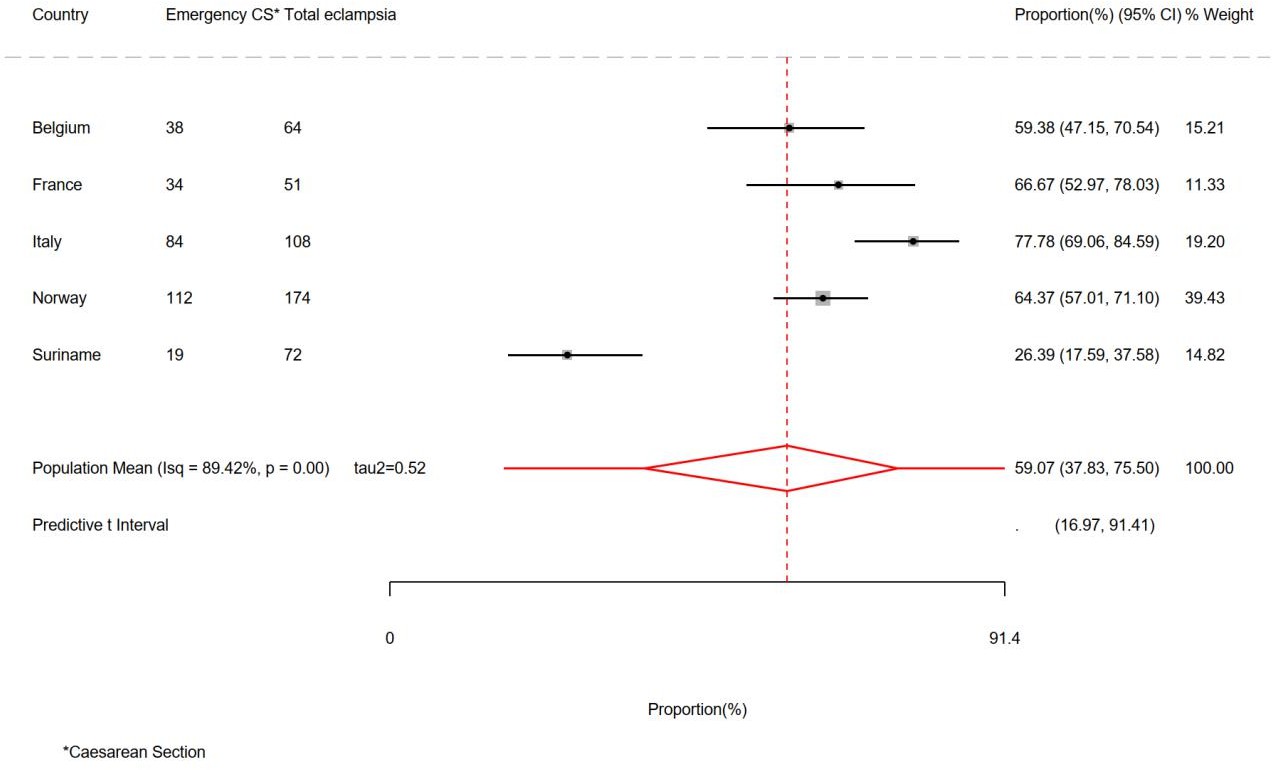
**

# Figure S18a. Mode of delivery: pooled proportion of antepartum among urgent/emergency CSs

**
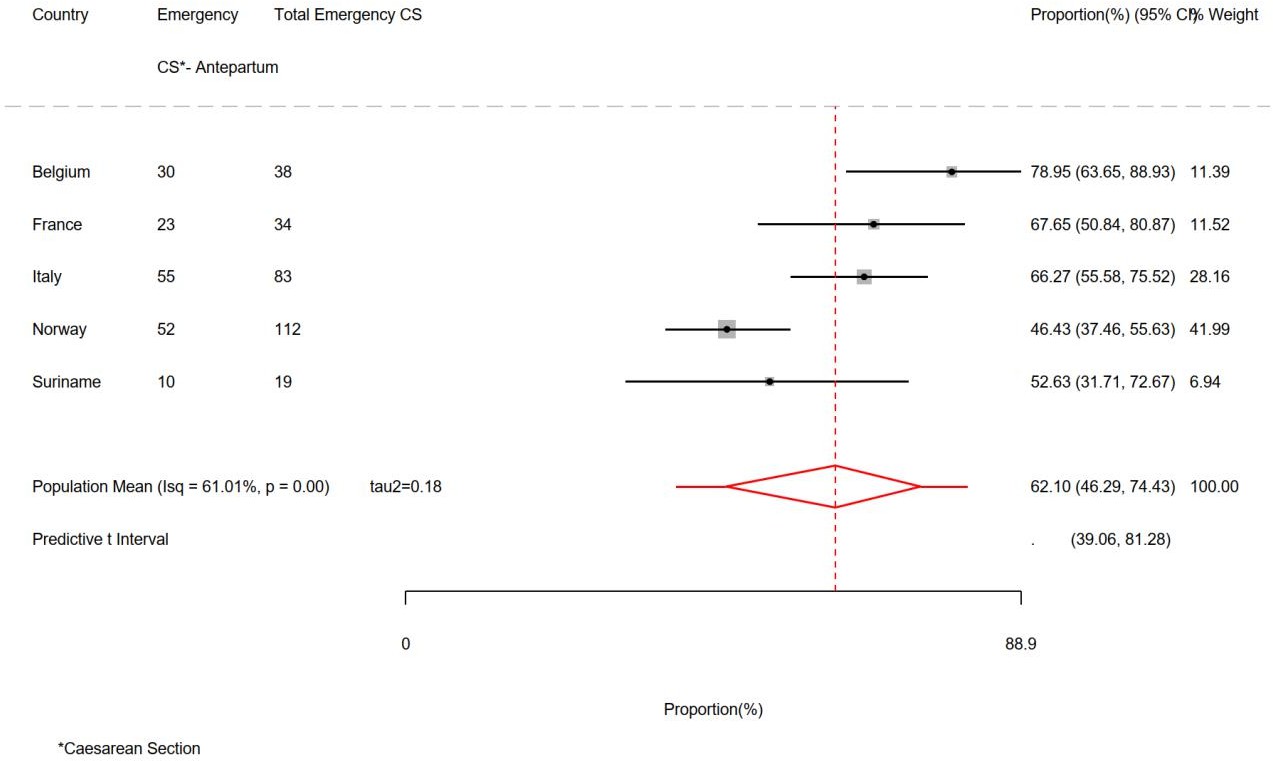
**

# Figure S18b. Mode of delivery: pooled proportion of intrapartum among urgent/emergency CSs

**
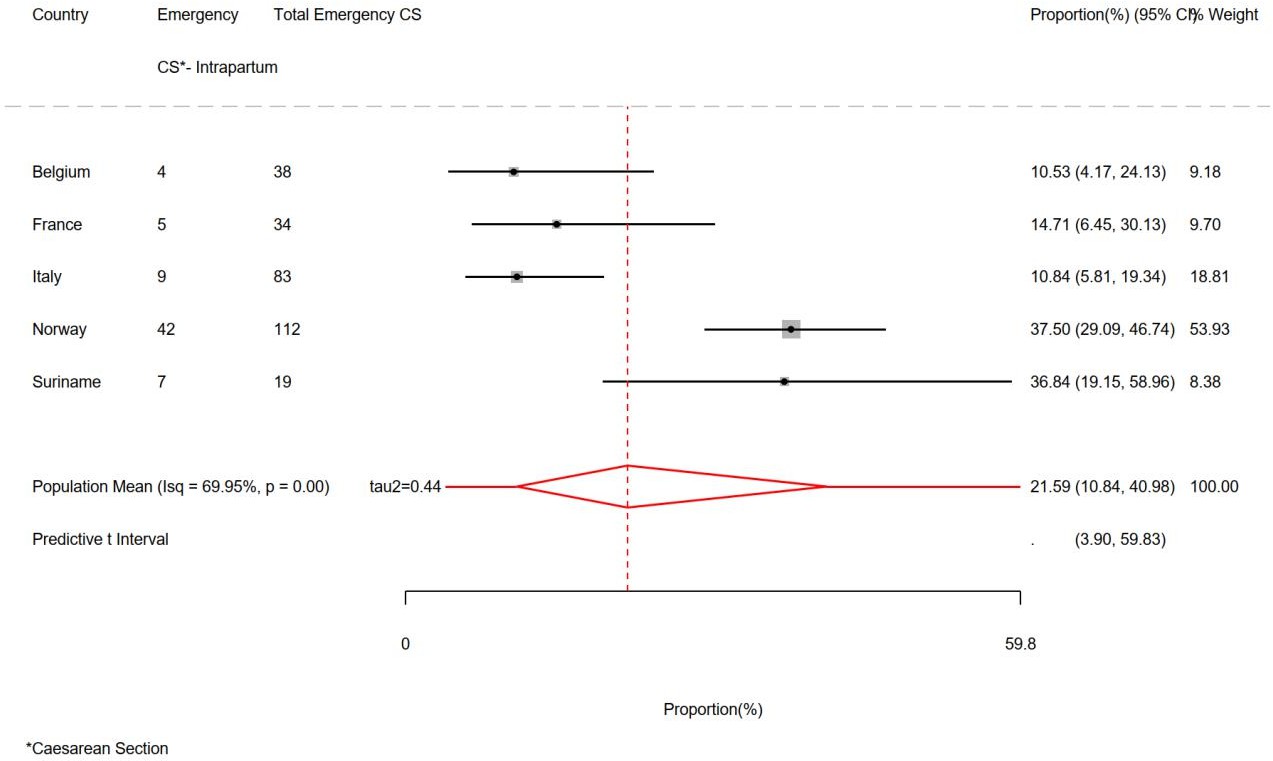
**

# Figure S19. Maternal outcomes: pooled proportion of preterm deliveries

**
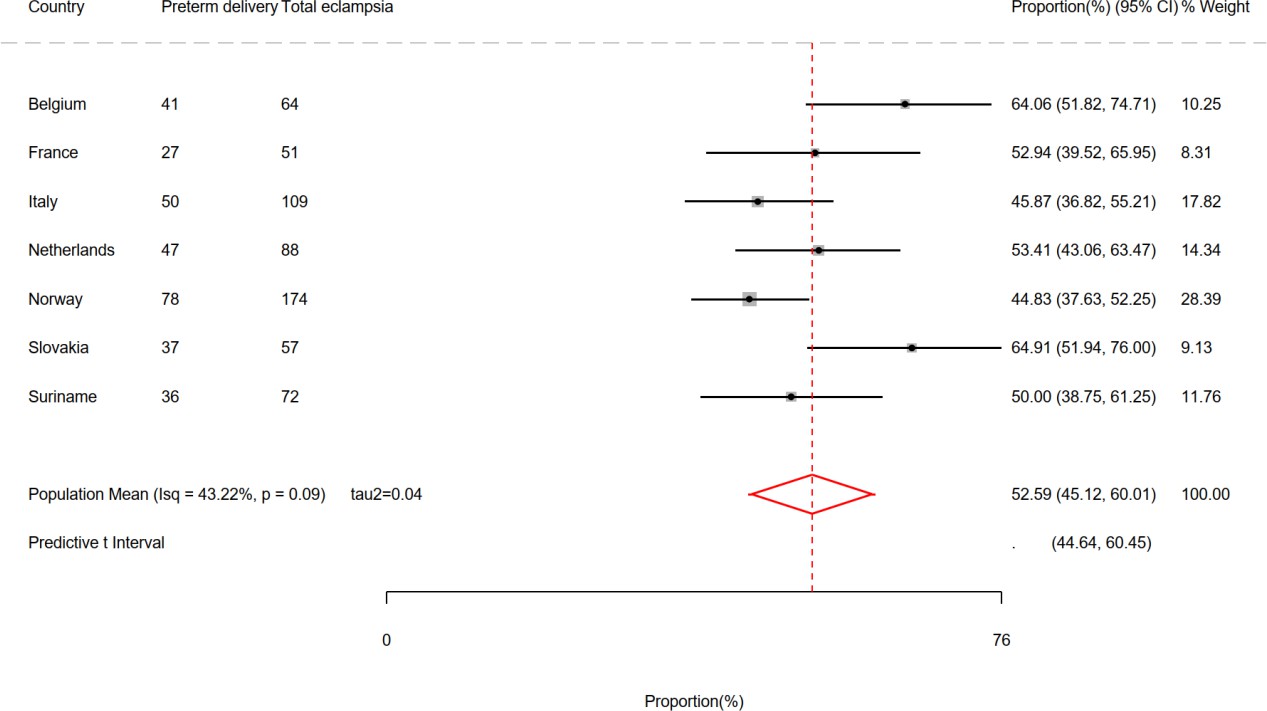
**

# Figure S19a. Maternal outcomes: pooled proportion of antepartum among preterm deliveries

**
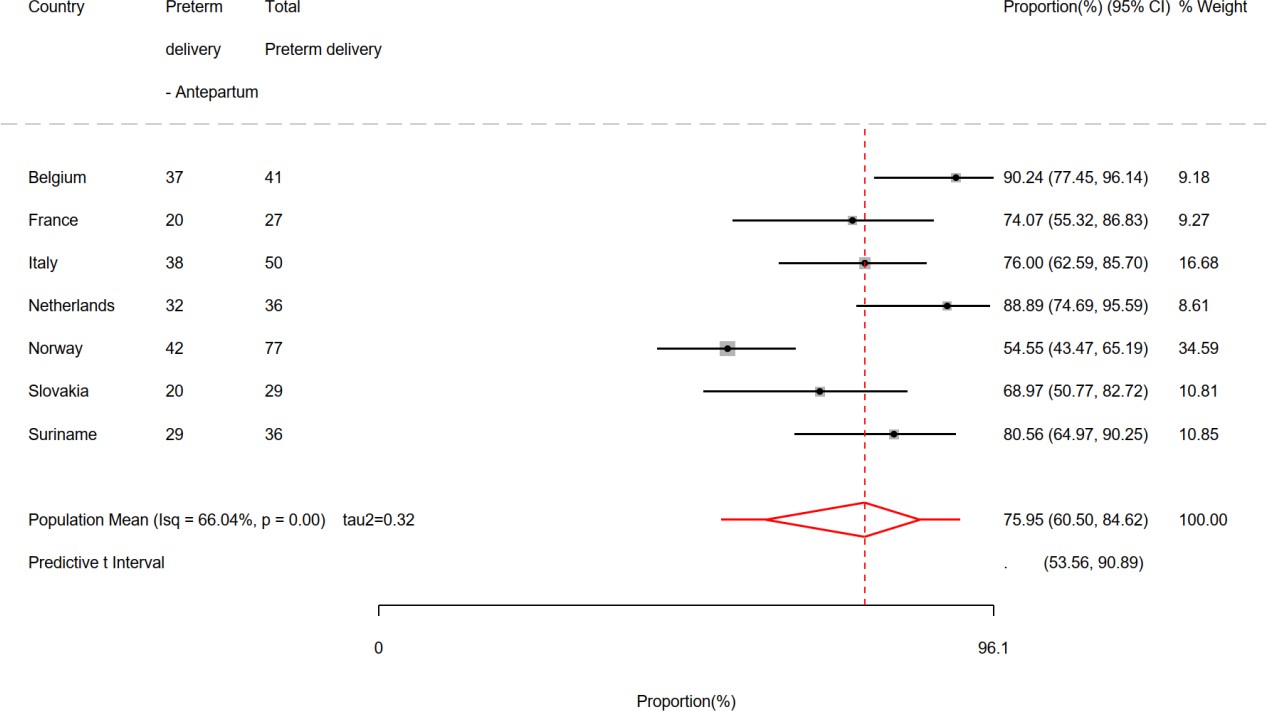
**

# Figure S19b. Maternal outcomes: pooled proportion of intrapartum among preterm deliveries

**
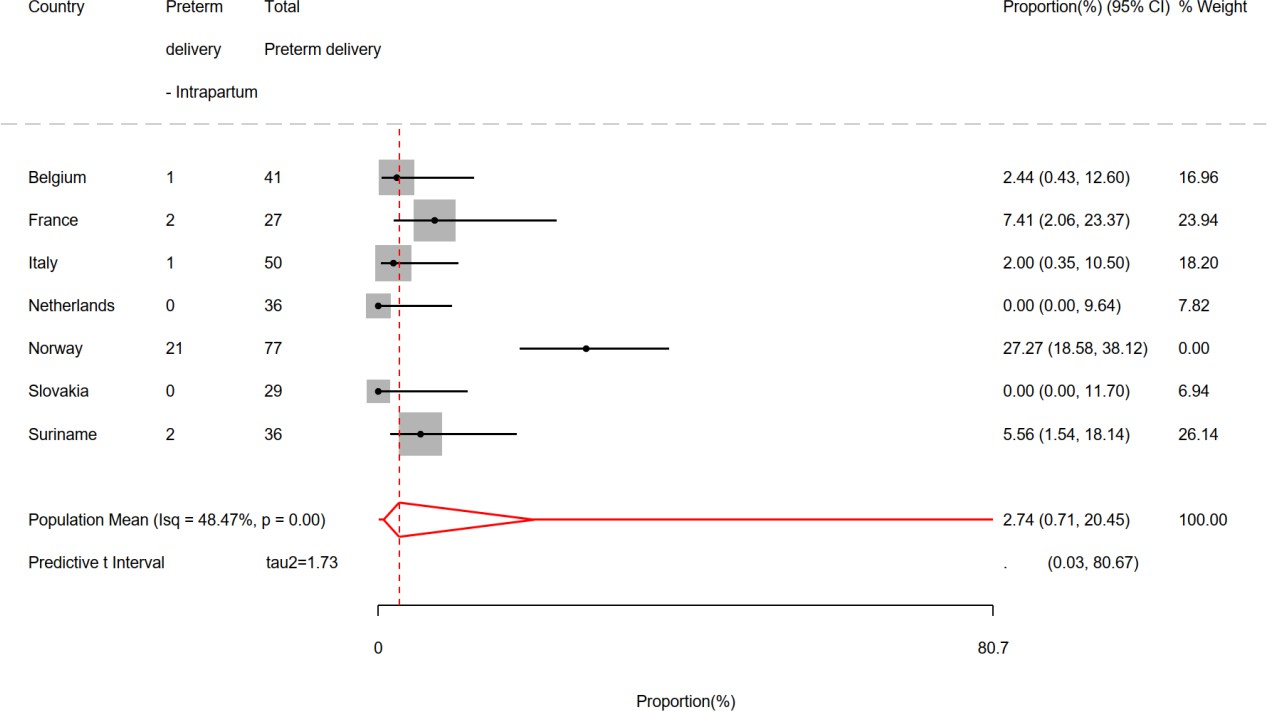
**

# Figure S19c. Maternal outcomes: pooled proportion of postpartum among preterm deliveries

**
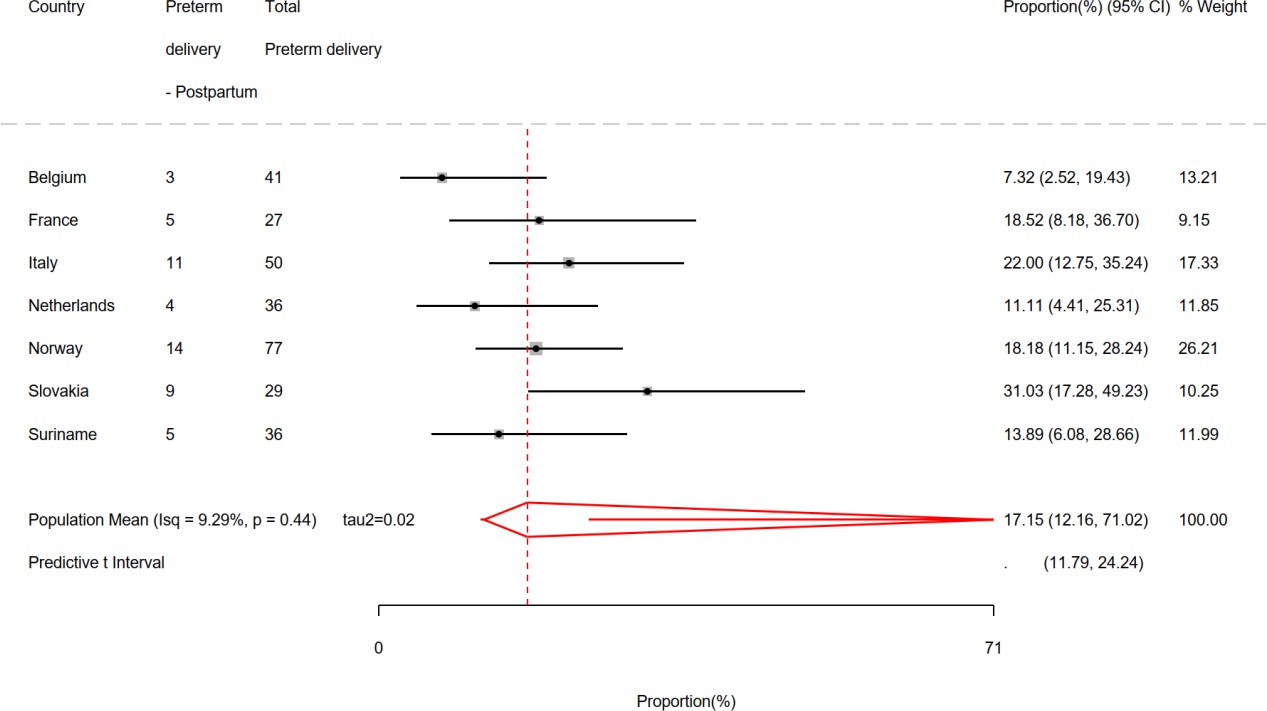
**

# Figure S20. Maternal outcomes: pooled proportion of higher-level monitoring admission

**
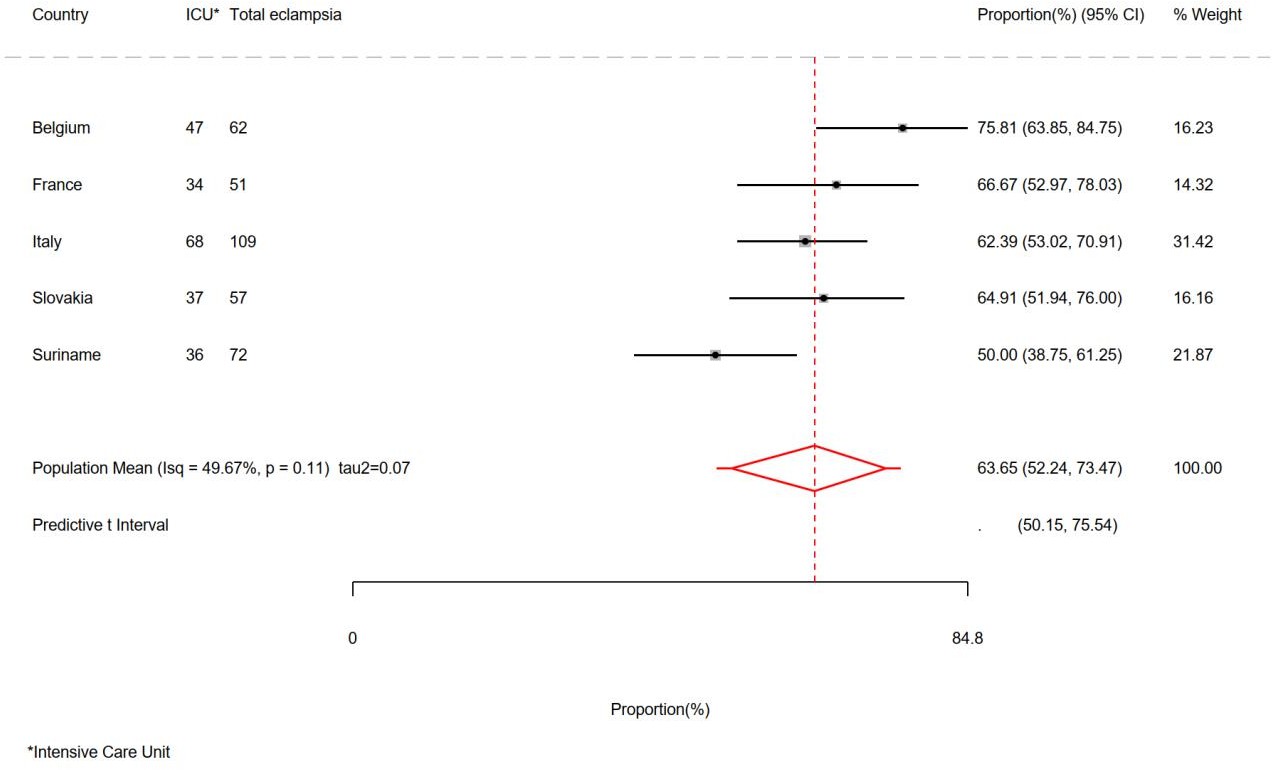
**

# Figure S20a. Maternal outcomes: pooled proportion of higher-level monitoring admission (restricted to HICs)

**
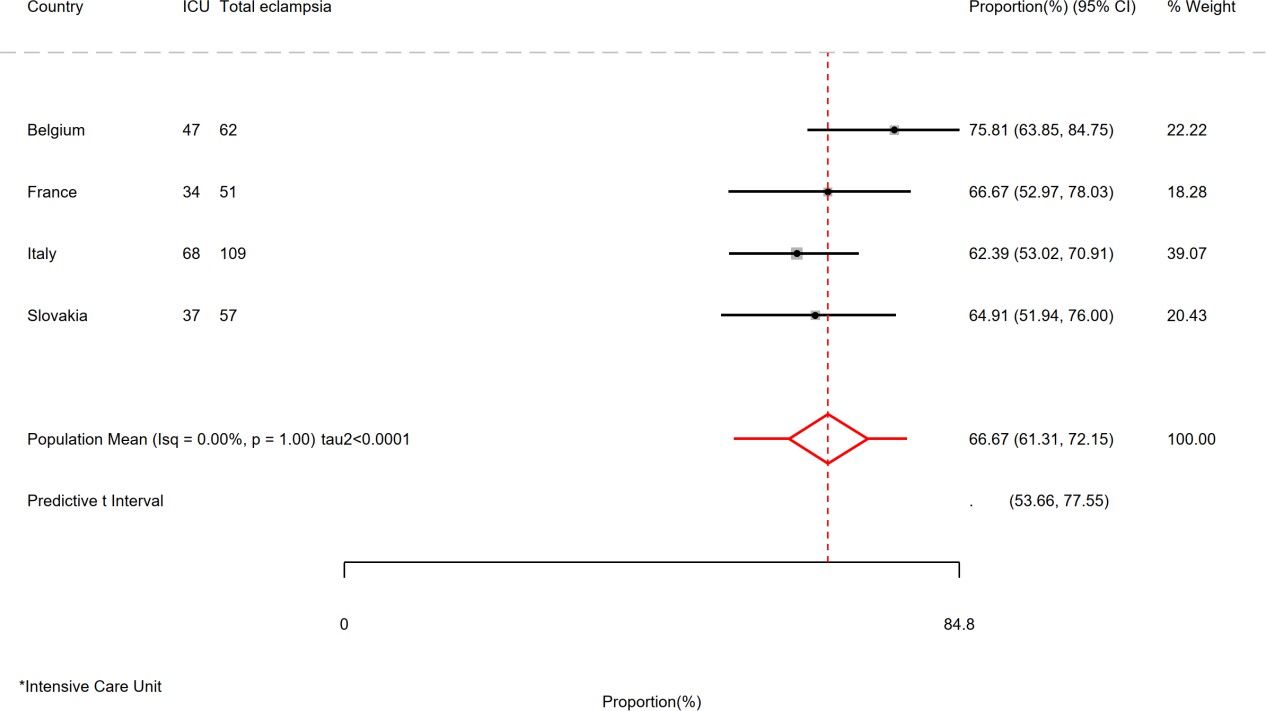
**

#
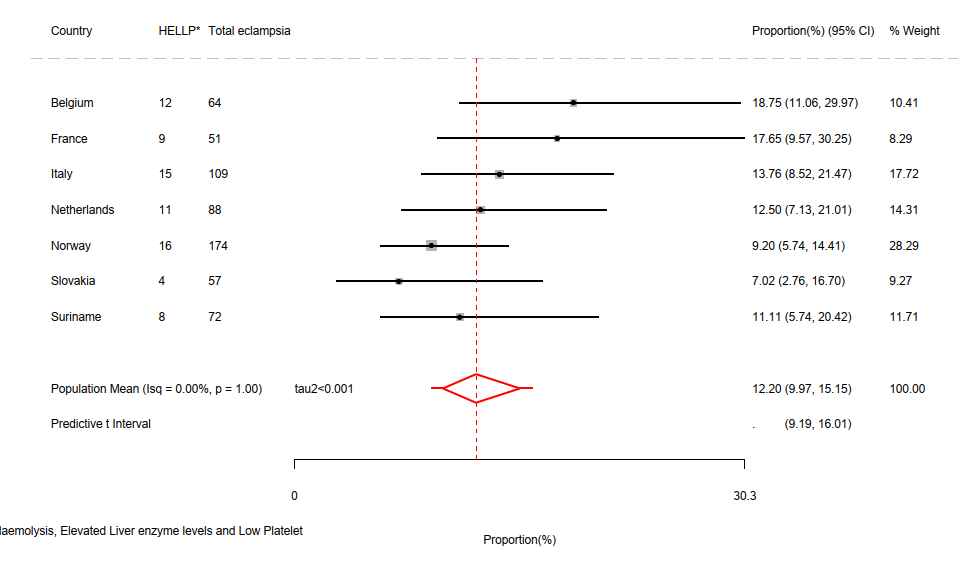
Figure S21. Maternal outcomes: pooled proportion of HELLP syndrome

# Figure S22. Maternal outcomes: pooled proportion of PRES (restricted to HICs)

**
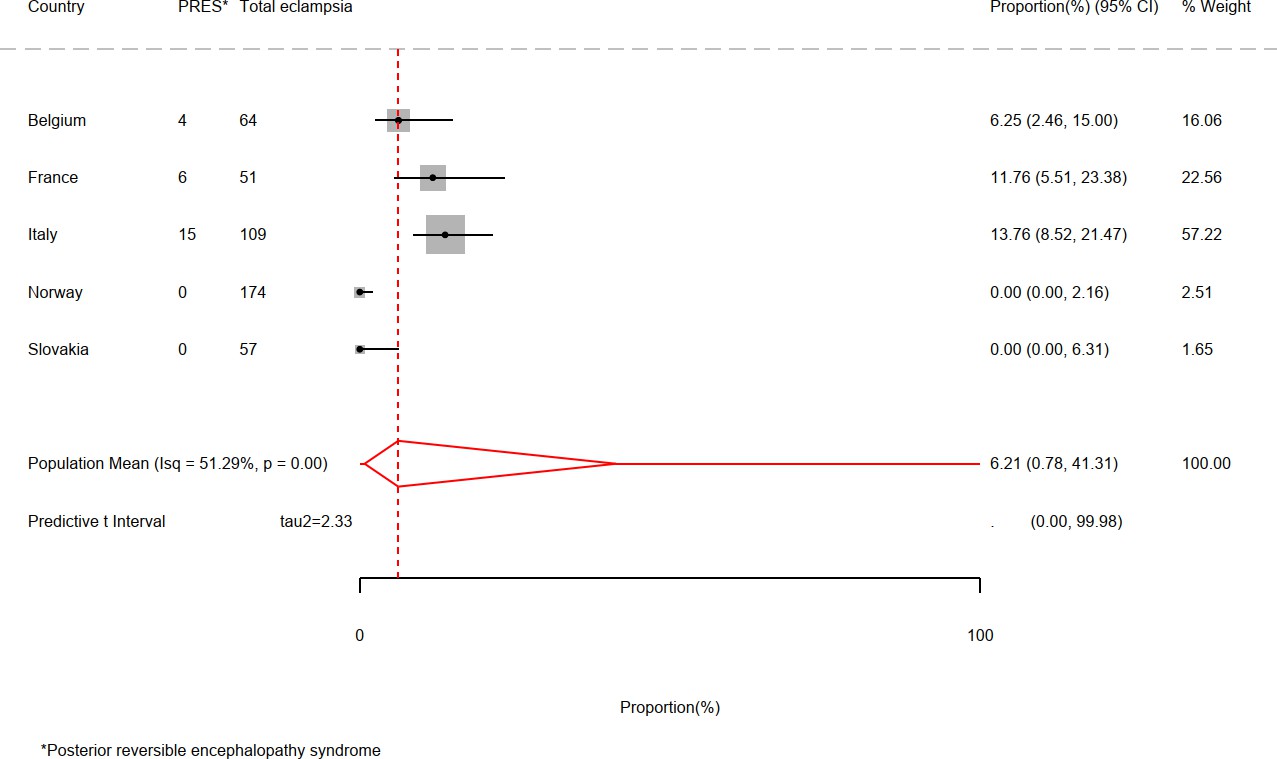
**

# Figure S23. Neonatal outcomes: pooled proportion of Apgar 5th <7

**
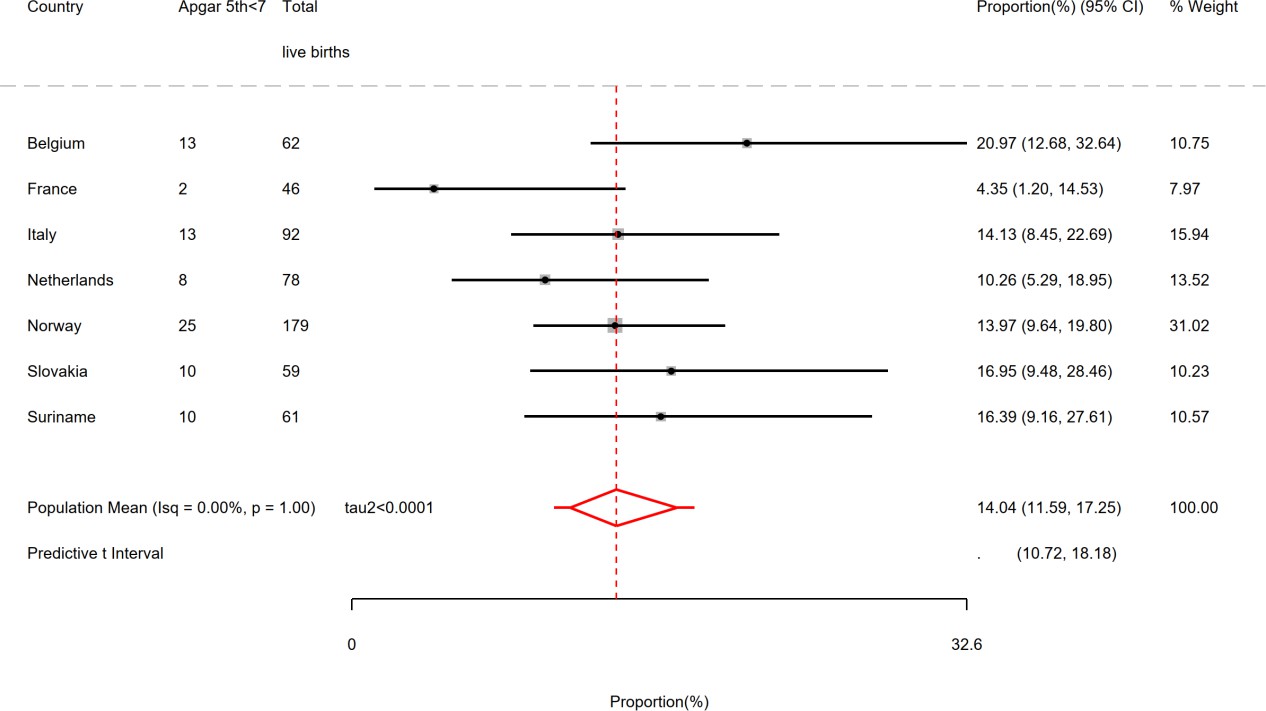
**

# Figure S24. Neonatal outcomes: pooled proportion of NICU admission

**
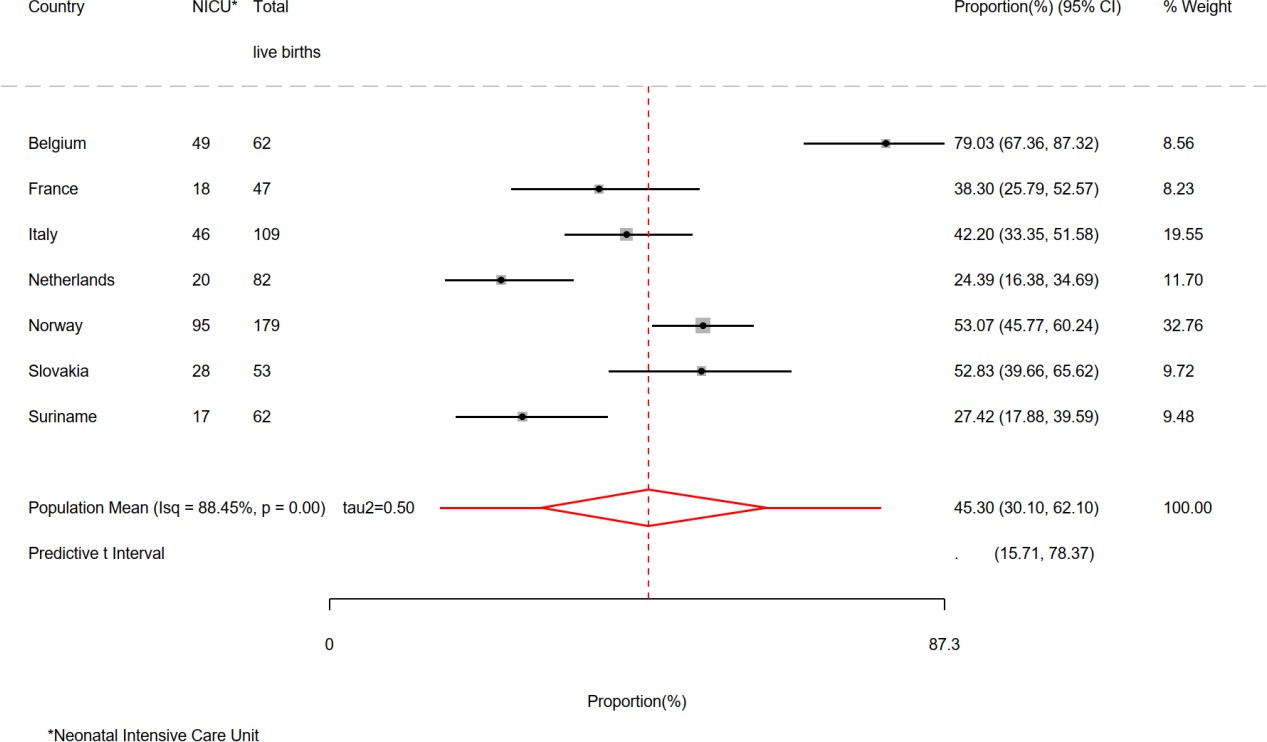
**

# Figure S24a. Neonatal outcomes: pooled proportion of NICU admission (restricted to HICs)

**
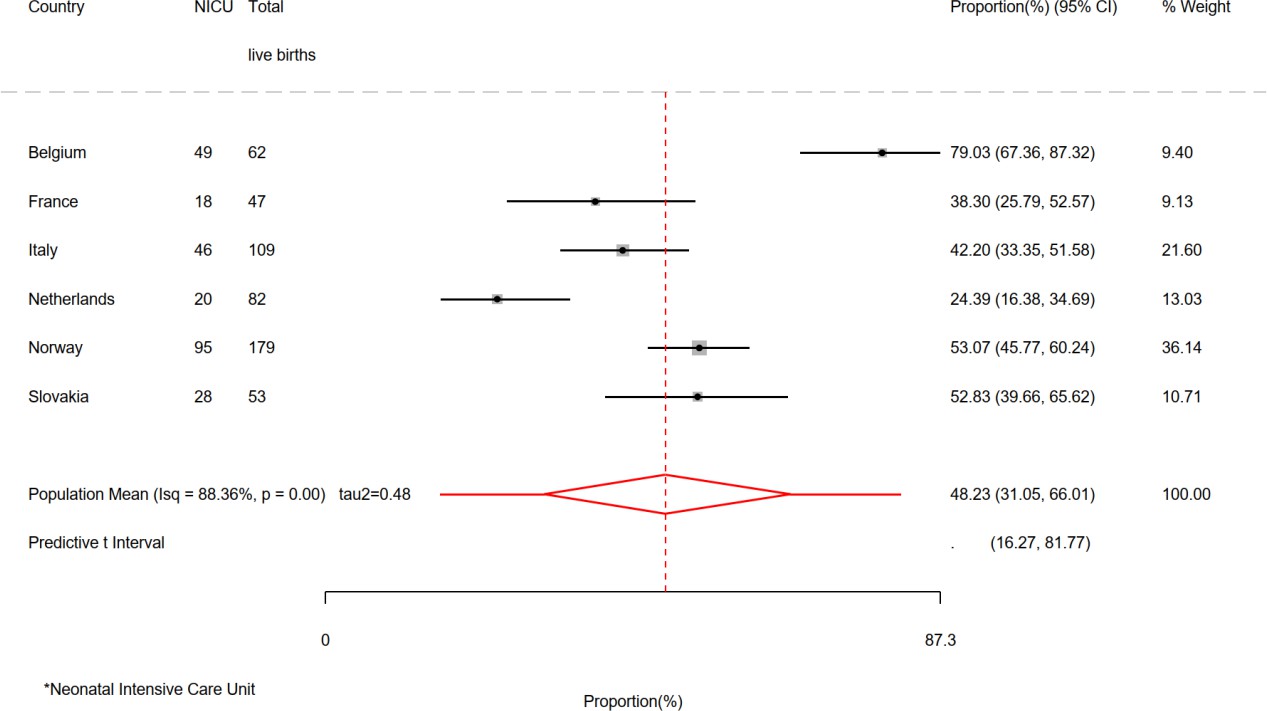
**

# References

1. Langedock A, Vandenberghe G, Leeuw V, et al. Eclampsie in België: resultaten van het Belgian Obstetric Surveillance System. *Gunakeya* 2019; **24**: 6-12.
2. Verschueren KJC, Paidin RR, Broekhuis A, et al. Why magnesium sulfate 'coverage' only is not enough to reduce eclampsia: Lessons learned in a middle-income country. *Pregnancy Hypertens.* 2020; **22**: 136-

43. DOI: 10.1016/j.preghy.2020.08.003.

1. Knight M, UKOSS. Eclampsia in the United Kingdom 2005. *BJOG.* 2007; **114**: 1072-8. DOI: 10.1111/j.1471-0528.2007.01435.x.
2. Schaap T, Bloemenkamp K, Deneux-Tharaux C, et al. Defining definitions: a Delphi study to develop a core outcome set for conditions of severe maternal morbidity. *BJOG* 2019; **126**: 394-401. DOI: 10.1111/1471-0528.15515.
3. Europeristat. European Perinatal Health Report. Core indicators of the health and care of pregnant women and babies in Europe from 2015 to 2019. 2022. [https://www.europeristat.com/images/Euro-](https://www.europeristat.com/images/Euro-Peristat_Fact_sheets_2022_for_upload.pdf) [Peristat_Fact_sheets_2022_for_upload.pdf.](https://www.europeristat.com/images/Euro-Peristat_Fact_sheets_2022_for_upload.pdf) (Accessed February 3, 2025).
4. Verschueren KJC, Prüst ZD, Paidin RR, et al. Childbirth outcomes and ethnic disparities in Suriname: a nationwide registry-based study in a middle-income country. *Reprod Health.* 2020; **17**: 62. DOI: 10.1186/s12978-020-00917-4.
5. Diguisto C, Saucedo M, Kallianidis A, et al. Maternal mortality in eight European countries with enhanced surveillance systems: descriptive population based study. *BMJ* 2022; **379**: e070621. DOI: 10.1136/bmj-2022-070621.
6. Vandenberghe G, Roelens K, Van Leeuw V, Englert Y, Hanssens M, Verstraelen H. The Belgian Obstetric Surveillance System to monitor severe maternal morbidity. Facts Views Vis Obgyn. 2017 Dec;**9**(4):181-8.
7. Korb D, Azria E, Sauvegrain P, et al. Population-based study of eclampsia: Lessons learnt to improve maternity care. *PlosONE* 2024; **19**: e0301976.
8. Maraschini A, Salvi S, Colciago E, et al. Eclampsia in Italy: A prospective population-based study (2017- 2020). *Pregnancy Hypertens* 2022; **30**:204-209.
9. Schaap TP, van den Akker T, Zwart JJ, van Roosmalen J, Bloemenkamp KWM. A national surveillance approach to monitor incidence of eclampsia: The Netherlands Obstetric Surveillance System. *Acta Obstet Gynecol Scan.* 2019; **98**: 342–350. DOI: 10.1111/aogs.13508.
10. Engjom HM, Morken NH, Høydahl E, Norheim OF, Klungsøyr K. Risk of eclampsia or HELLP- syndrome by institution availability and place of delivery - A population-based cohort study. *Pregnancy Hypertens.* 2018; **14**: 1-8. DOI: 10.1016/j.preghy.2018.05.005., 2018
11. McCullough L, Korbeľ M, Daniš J, et al. Analysis of eclampsia cases in the Slovak Republic in 2012- 2018. *Gynekol prax* 2022; **20**: 105 – 8.
